# Mental load in women and mothers: Causes of distress, personality traits, and psychopathology

**DOI:** 10.1371/journal.pone.0356255

**Published:** 2026-09-02

**Authors:** Stefanie M. Jungmann, Stella F. Garbe Huedo, Karlotta A. S. Jacobs, Nina K. Pothorn

**Affiliations:** Department of Clinical Psychology and Psychotherapy of Childhood and Adolescence, Johannes Gutenberg-University Mainz, Mainz, Germany; Universita Cattolica del Sacro Cuore Sede di Roma, ITALY

## Abstract

Mental load has received increasing public attention, particularly following societal changes such as the COVID-19 pandemic and shifts toward greater gender equality. However, despite its societal relevance, it remains comparatively under-researched. Following a literature review on mental load, two cross-sectional online survey studies systematically examined the burden of invisible (mental load) versus visible household and caregiving work, the causes of this burden, and associations with personality traits and psychological symptoms (affect, anxiety, depressiveness). Participants were recruited online via university mailing lists, social media, and parent forums. In Study 1 (*N* = 368 women; M = 35.98 years, SD = 8.87), it was found that mothers (vs. women without children) perceived invisible tasks such as planning or organizing significantly more stressful and unequally distributed compared to visible tasks. Mental load was significantly negatively related to positive affect and significantly positively related to negative affect, anxiety, and depressive symptoms. In both studies, constantly thinking about tasks and the perception that tasks never appear to be completed were cited as the most common causes of mental load. The results of Study 2 (*N* = 1891 mothers; M = 38.18 years, SD = 5.18) showed that mental load is positively related to neuroticism and conscientiousness, with the association of mental load and neuroticism moderated by being a single mother. Practical implications at the individual, couple, and societal levels are derived from these findings.

## Introduction

When thinking of family, domestic and care work, the first thing that usually comes to mind are the visible tasks such as cooking, doing laundry, or driving children to their leisure activities. However, these visible tasks are constantly accompanied by invisible cognitive processes such as planning, organizing, and relational/emotional work. Despite increasing gender equality on average, at least 75% of unpaid domestic and care work (visible and invisible) is done by women and mothers [1,2]. Beyond these observable activities lies a largely unseen layer of ongoing thinking work: remembering, anticipating, monitoring, coordinating, and emotion/relationship management. In everyday life, this’mental load’ can be experienced as intrusive and difficult to switch off, because it is boundaryless and recurrent [3]. The invisible processes or cognitive work have received increasing attention in the media and society in recent years (e.g., during the COVID-19 pandemic), but are scientifically under-researched, including clear conceptualizations, correlates, and mechanisms [3–5].To address these gaps, the present manuscript first differentiates key concepts (mental labor vs. mental load), summarizes existing evidence on mental health correlates and stressful characteristics, and then reports two empirical studies that (a) compare perceived burden of invisible vs. visible tasks, (b) identify the most frequently endorsed reasons for the burden, and (c) examine associations with psychological symptoms and personality traits. In the following Literature Review, we synthesize prior work on mental load and related concepts, with a focus on its stressful characteristics and associations with mental health and personality. We then derive the aims and hypotheses of the present research before detailing the Methods.

## Literature review

### Descriptions and conceptualizations of mental labor and mental load

The labels and conceptualizations of these invisible cognitive processes in the context of domestic and care work are not used consistently. In contrast to visible or physical labor, the terms *cognitive dimension*, *cognitive labor*, *cognitive load*, *mental labor*, and *mental load* exist to describe these non-visible cognitive aspects [3–5]. The terms originate from cognitive science and occupational psychology. Descriptions of *cognitive or mental labor* in the context of home and care work have only been added in recent years and mostly include cognitive processes such as thinking, planning (including anticipating and identifying), organizing, and managing (including decision-making and monitoring) in everyday life at home [5,6]. Robertson et al. [4] identified six aspects of mental labor from interviews with mothers: (a) planning and developing strategy, (b) anticipating and monitoring, (c) meta-parenting (attitudes and intentions), (d) learning and remembering, (e) overall management, and (f) self-regulation (themselves and others). In particular, care work is usually accompanied by emotional work (e.g., emotion regulation and expression), so *mental load* is assumed to result from a combination of cognitive and emotional load [3]. Alternatively, *mental load* has also been commonly referred to as the costs or resulting burden of mental labor, and some descriptions also include the burden of responsibility as a cause of load [5,7]. For the studies presented here, we define *mental load (ML)* as burden associated with the non-visible, cognitive processes of thinking about, planning, organizing, and managing, and relational and emotional work in the context of everyday unpaid housework and caregiving.

### Mental labor and mental health

Previous studies found that mental labor negatively impacts well-being and mental health. Ciciolla and Luthar [8] showed in a survey of 393 mothers that feeling disproportionate responsibility for family management is associated with lower well-being and relationship satisfaction. Another study of mothers [9] found that higher perceived effort and lower perceived reward of housework and care work are associated with poorer health, more physical symptoms, and more anxiety and depressive symptoms. An unfavorable balance of effort and reward was thereby better able to explain mental health than physical health in mothers [9]. The stresses on women and mothers were also particularly evident in the COVID-19 pandemic. During the COVID-19 pandemic (particularly during the lockdown), much of the double burden (housework, care work including homeschooling, and paid work, respectively) fell on women and mothers, who reported increased levels of burnout, loneliness, anxiety, and depression during this time [10,11].

### Stressful characteristics of mental labor

Dean et al. [3] describe three characteristics, in particular, that contribute to the stressful and exhausting nature of mental labor: 1. these tasks and duties are ‘invisible’ (e.g., little valued), 2. they are boundaryless (e.g., flowing or interfering with falling asleep, paid work, or leisure), 3. they are ongoing (e.g., few short- or longer-term breaks). These characteristics are supported by the Transactional Stress Model [12], which states that the subjective evaluation of a situation/event as stressful is crucial, as are the individual resources available to cope with the situation. In addition, emotional work in the context of the family is also a stress factor, similar to what studies in certain service professions have shown [3,13]. Mikolajczak et al. [14] refer to parental burnout, which highlights an imbalance between perceived stressors and resources and associated psychological exhaustion in the context of parental care and responsibilities. Some previous studies found that cognitive family work is associated with not being able to switch off, exhaustion, negative affect, irritability, and sadness, and these psychological symptoms also negatively affect paid work and partnership quality [5,6,15]. Overall, some characteristics of mental work that cause strain and thus mental load are mentioned in the literature, such as the (sole) sense of responsibility, perceived unequal distribution between partners, time expenditure and chronicity, overlap on other areas of life, and the emotional component [3,5,16]. A recent systematic review [17] identified 26 articles on parental burnout and categorized associated factors according to the Ecological Systems Theory (EST) [18]. In particular, factors at the individual microsystem level, e.g., sociodemographic data, personality factors, and anxiety/depression, were found to be associated with parental burnout. The latter (mental health problems) e.g., due to the overlap of dysfunction of the hypothalamic-pituitary-adrenal axis (stress axis). The (risk) factors associated with parental burnout may resemble those linked to mental workload. However, there is a lack of systematic scientific research examining why mental labor is perceived as stressful and how mental load relates to mental health. Gaining a precise understanding of the underlying processes and reasons why mental labor can be subjectively experienced as highly burdensome, as well as of its exact connection to mental health, is essential for developing effective strategies to reduce the burden of invisible work and promote mental well-being.

### Individual and social factors influencing mental load

In addition, there are a few descriptions of which individual and social factors can influence this burden. Lee and Waite [19] found that gender differences (to the disadvantage of women) in household workload increased significantly when cognitive work was included; and particularly (or only) in women (versus men) cognitive work was found to have a negative impact on mental health [6,15]. In the context of parental burnout, Piotrowski et al. [20] found that the personality traits conscientiousness (ß = −.15) and emotional stability (ß = −.32) significantly accounted for additional variance in emotional burnout beyond sociodemographics. A meta-analysis [21] found that work-family conflict (taking one role complicates the other role) mediates the association between personality traits such as agreeableness, conscientiousness, and neuroticism in particular, and mental health. This is also consistent with findings that the personality traits neuroticism and conscientiousness are most strongly related to well-being and mental health [22,23]. For the area of mental labor and mental load in the context of household and care work, the correlations with personality traits have hardly been studied so far. Investigating the relationship between personality traits and mental load is important because certain personality factors, as described here, may act as risk factors for experiencing high levels of strain in the context of invisible work. Moreover, personality traits may interact with mental load in ways that intensify or mitigate the subjective perception of burden. Identifying such traits, and understanding both their independent effects and their interaction with mental load, can inform targeted prevention strategies, which in turn may help to reduce the burden of invisible work and the associated stressors.

### The present studies

Mental labor can be particularly stressful for women, can be associated with poorer mental health, and may have negative consequences for various domains of life (e.g., partnership and family relationships, mental health symptoms, sleep quality, and even paid employment) [8,9,16,24]. In recent years, mental load has gained substantial attention in public discourse, reflecting not only its contemporary relevance but also its potential far-reaching implications across multiple domains of life, including family relationships, mental health, and occupational functioning. Despite its societal importance and the breadth of its possible consequences, mental load has received remarkably little systematic investigation within the scientific literature [24]. Given the scarcity of empirical research on mental labor, particularly with regard to its underlying mechanisms and determinants, it is crucial to systematically investigate which specific aspects of mental labor constitute the perceived burden, what role personality factors may play (e.g., as potential risk or protective factors), and how mental labor is related to psychopathological symptoms. Addressing these questions is essential, as a precise understanding of the stressors, risk factors, and their possible interactions provides the basis for developing evidence-based strategies aimed at reducing the burden of mental labor (e.g., through targeted prevention and intervention measures). By focusing on the identification of risk factors, as well as the associations between mental labor/load, personality traits, and mental health symptoms, this study seeks to address important gaps in the literature and contribute to a more comprehensive understanding of the phenomenon.

This article comprises two consecutive studies. Both studies systematically examined the perceived burden of ‘visible’ (physical) versus ‘invisible’ (cognitive/mental labor) tasks in the family context, along with the reasons underlying this burden (with Study 2 conducted in a larger sample for confirmation). Given the characteristics of mental load and its overlap with parental burnout [3,5,14,16–18], it was assumed that ‘invisible’ tasks would be perceived as more burdensome than ‘visible’ tasks (hypothesis 1). Furthermore, the studies aimed to identify the specific reasons and characteristics that make mental labor burdensome. In particular, the investigation sought to confirm both the characteristics proposed in the model by Dean et al. [3] and those empirically identified to date (e.g., responsibility, time commitment) [3,5,16] (hypothesis 2).

In Study 1, the focus is additionally on examining the relationship between mental load and psychological symptoms (anxiety and depressive symptoms). Women and mothers experiencing high levels of overall strain (e.g., during the COVID-19 pandemic) frequently report elevated levels of burnout, loneliness, and symptoms of anxiety and depression [10,11,25]. Such findings are consistent with biological and psychological models (see above: HPA axis, Transactional Stress Model [12]) and with previous research demonstrating that mental labor is associated with exhaustion/excessive demands, negative affect [16], and, in more recent work, depressive symptoms [24]. Against this background, it is hypothesized that medium-strength positive correlations will be found between mental load (perceived burden of ‘invisible’ tasks) and symptoms of anxiety and depression (hypothesis 3 and 4).

In Study 2, the focus is additionally on the relationship between the personality traits neuroticism as well as conscientiousness and mental labor in the context of family (traits, that are particularly relevant in the context of parental burnout, see above [20]). Individuals with higher neuroticism are considered to be more emotionally unstable, sensitive, and tend to become more easily unbalanced under stress [26]. In the context of parental burnout, there is also the assumption that neuroticism is associated with deficits in emotion regulation and that emotionally unstable parents react more strongly to life events, which can increase the likelihood of parental burnout [17]. For example, Le Vigouroux et al. [27] found that a high degree of neuroticism is a risk factor for parental burnout. So similar to findings in burnout and stress research [17,20,28,29], it could also be hypothesized that neuroticism is positively related to mental load (hypothesis 5). Regarding conscientiousness, individuals with higher levels are thought to be more organized, disciplined, and have better time management [20], and negative associations have been found between conscientiousness and parental and job burnout [20,28]. A previous study showed that low conscientiousness is a risk factor for parental burnout [27]. Accordingly, a negative correlation between conscientiousness and mental load in the context of family was assumed (hypothesis 6). Research consistently shows that single parents, especially single mothers, experience substantially higher levels of psychological distress, depressive symptoms, and burnout compared to partnered parents [10,11,25,30]. In parallel, personality research has identified neuroticism and conscientiousness as predictors of parental burnout and related forms of strain [17,27]. It seems plausible that the combination of these personality traits with the already high demands faced by single parents could further intensify the perceived burden of mental load. Based on this evidence, being a single parent was hypothesized to moderate the associations between neuroticism/conscientiousness and mental load (hypothesis 7). Since most of the mental work in the field of domestic and care work is done by women and mothers [16], above hypotheses were investigated on women and mothers.

#### Hypotheses.

In the following, we summarize the study hypotheses (H1-H7).

H1: Invisible tasks (mental labor) are perceived as more burdensome and less equally distributed than visible tasks, particularly among mothers.

H2: The most frequently endorsed reasons for mental load reflect key stress characteristics of mental labor such as ongoing/never finished, boundaryless/constant thinking, low appreciation, responsibility, and unequal distribution.

H3: Mental load is positively associated with symptoms of generalized anxiety.

H4: Mental load is positively associated with depressive symptoms.

H5: Mental load is positively associated with neuroticism.

H6: Mental load is negatively associated with conscientiousness.

H7: Single parent status moderates the associations between neuroticism/conscientiousness and mental load.

## Study 1

### Method

#### Participants.

Potential participants for the Online survey ‘Mental Load in Families 2022’ were recruited (October 24 – November 26, 2022) via university email distribution lists, social media (e.g., Twitter), and parent forums to maximize heterogeneity. Inclusion criteria were an age of at least 18 years and that the persons live with a partner and/or at least one child in a common household. Completion of the online survey took approximately 15 minutes. Participants could voluntarily enter a raffle to win one of three €15 bookstore vouchers (or psychology students could receive 0.5 course credits). A required sample size of at least *N* = 88 was calculated a priori (G* Power 3.1.9.7 [31] correlation, one tail: ρ = 0.3, α = 0.05, 1-β = 0.90; mixed ANOVA *N* = 62, f = 0.25, α = 0.05, 1-β = 0.90, correlation among measures = 0.3). 412 individuals took part in the survey (89.3% female, 10.7% male, 0% diverse/non-binary; *M* = 36.39, *SD* = 9.18 years). Because the hypotheses referred only to women, *N* = 368 women entered the analyses (*M* = 35.98, *SD* = 8.87 years; 59% university degree). *N* = 263 (71.5%) women have children, per woman average *M* = 1.33 (*SD* = 1.08) children with an average age of children of *M* = 7.23 (*SD* = 5.17) years. 54.6% reported living with married partner and at least one child, 31.3% with partner, 7.3% with unmarried partner and at least one child, 3.8% as a single mom with at least one child, 3.3% in a patchwork family, 0.5% with partner and at least one foster child, 0.3% same-sex couple with at least one child, 2.7% other/supplementary (a few people made multiple responses). In terms of employment status, 56.3% are employees, 22.6% are students, 6.3% are self-employed, 4.9% are on parental leave, 1.6% are in vocational training, 1.1% are housewives, and 0.3% each are job seekers or on early retirement. The ethics committee of the Psychological Institute of the Johannes Gutenberg-University Mainz has approved the study project. All participants provided written informed consent prior to participation, including consent for the scientific use of their anonymized data. Data were processed in accordance with applicable data protection regulations.

#### Measures.

To understand what we mean by ‘invisible’ and ‘visible’ tasks in home and care work, we gave a few descriptions and examples at the beginning of the study: ‘Mental Load refers to stresses caused by frequent, ‘invisible’ cognitive processes of thinking about and organizing and responsibilities in daily life. To better examine different processes and tasks, we distinguish 1. ‘invisible’ tasks: processes of planning/organizing (such as cooking preparations: who likes/doesn’t like to eat what, where/when/what to buy, etc.) and 2. ‘visible tasks’: performing the task (cooking itself), but of course these are clearly interrelated in everyday life.“

**Unpaid/paid work, ‘invisible’/’visible’ unpaid work in the field of household and care work, burden and causes of burden:** We asked 1) the number of hours per week of paid work of the women and their partners, 2) the number of hours per week of unpaid work (housework and care work), subdivided into 2a) ‘invisible’ and 2b) ‘visible’ tasks as well as 3) the distribution between partners in % and 4) the satisfaction with this distribution. Regarding the burden, it was asked 5) how burdensome the ‘invisible’ and ‘visible’ tasks are perceived (0–100, 100 very burdensome) and 6) what exactly they perceived as stressful about the 6a) ‘invisible’ and 6b) ‘visible’ tasks using predefined multiple-choice response options (such as find nothing stressful about it, constant thoughts about it for ‘invisible’ tasks, amount/frequency, intensity/complexity/physical demands for ‘visible’ tasks, uneven distribution, low/lack of appreciation, lack of pay, etc.) and an additional open-text ‘Other’ category. Responses entered in the ‘Other’ category were reviewed and summarized into overarching categories where appropriate prior to analysis.

**Affect, anxiety, and depressive symptoms:** The Positive and Negative Affect Schedule – short version (PANAS-short [32]) measures positive and negative affect with 5 items each (adjectives such as ‘enthusiastic’ and ‘nervous’, 5-point Likert scale: 1 = very slightly/not at all, 2 = a little, 3 = moderately, 4 = quite a bit, 5 = extremely). In the present study, Cronbach’s alpha were α = .79 (positive affect) and α = .77 (negative affect).

As a short scale for Generalized Anxiety Disorder (GAD-7 [33]), 7 items assess generalized worry and anxiety over the past 2 weeks (e.g., ‘Worrying too much about different things’, 4-point Likert scale: 0 = not at all, 1 = several days, 2 = more than half the days, 3 = nearly every day). In this study, the GAD-7 showed good internal consistency (α = .84). The GAD-7 can be used as a screening tool for anxiety severity with cut-off scores; however, in this study, a dimensional approach was relevant.

Depressive symptoms (over the last two weeks) were assessed with the Patient Health Questionnaire for screening depression (PHQ-2 [34]), which is an ultrashort questionnaire with two items (‘little interest or pleasure in doing thing’ and ‘feeling, down, depressed, or hopeless’, 4-point Likert scale:0 = not at all, 1 = several days, 2 = more than half the days, 3 = nearly every day). In the present study, the PHQ-2 showed an internal consistency of α = .75. The PHQ-2 can be used as a screening tool for depressive symptoms with established cut-off scores (e.g., ≥ 3 indicating possible major depression); however, in this study, a dimensional approach was adopted, treating PHQ-2 scores as a continuous measure.

#### Statistical analyses.

For the statistical analyses, SPSS Statistics 23 [35] was used. Normality of the study variables was assessed using the Shapiro–Wilk test. As all study variables significantly deviated from a normal distribution, Spearman rank correlations were used to test the correlational hypotheses. In addition, paired t-tests were calculated to examine differences in the satisfaction with the distribution and load of ‘invisible’ versus ‘visible’ work, and a mixed ANOVA was conducted to additionally examine the effect of group (women with vs. without children), as these parametric procedures are generally considered robust to moderate violations of the normality assumption, particularly in larger samples, consistent with the Central Limit Theorem. Prior to conducting the mixed ANOVA, homogeneity of variances was assessed. As the within-subject factor comprised only two levels (visible vs. invisible work), the assumption of sphericity was inherently satisfied and therefore did not require formal testing. To minimize potential sources of bias, no missing values were permitted in the dataset, as the online survey required all items to be completed before submission. This ensured complete data for all variables without the need for imputation. Furthermore, to reduce the risk of error accumulation due to multiple testing, only the analyses necessary to address each specific hypothesis were conducted, and the number of statistical tests per hypothesis was kept to the minimum required. The statistical analysis plan, including the alpha level for significance testing (p < .05 for significance, p < .001 for high significance), was determined prior to data analysis. For the effect size Cohen’s *d* [36], the formula from Dunlop et al. [37] was used, which also includes the correlation of the variables.

### Results

#### Descriptive statistics and comparing paid vs. unpaid and ‘invisible’ vs. ‘visible’ household-/care work.

Table 1 summarizes the means and standard deviations (or percentages) of the variables studied. As reported by the women, they take on 2/3 (66.2%) of hours of paid work and about twice as many hours of unpaid household and care work (both visible and invisible) compared to their partners. Regarding unpaid household-/care work, they perform 84.2% of invisible tasks and 69.4% of visible tasks.

**Table 1:** Means (*M*) and standard deviations (*SD*) or percent (agreement with question) on the variables studied (^*^hours per week, ^**^hours per day) in Study 1.

Variables	*M*	*SD*	range	%
Hours of paid work women^*^	24.07	13.78	0–70	
Hours of paid work women’s partner^*^	36.36	12.51	0–74	
Hours of unpaid ‘invisible’ work women^**^	2.81	4.13	0–40	
Hours of unpaid ‘invisible’ work women’s partner^**^	1.16	2.89	0–40	
Hours of unpaid ‘visible’ work women^**^	4.04	3.95	0–35	
Hours of unpaid ‘visible’ work women’s partner^**^	2.05	2.63	0–35	
Responsibility (%) of unpaid ‘invisible’ work (0–100%)			10–100	83.19
Responsibility (%) of unpaid ‘visible’ work (0–100%)			0–100	68.35
satisfaction with the distribution ‘invisible’ work (0–100)	44.01	28.88	0–100	
satisfaction with the distribution ‘visible’ work (0–100)	52.93	27.71	0–100	
perceived burden of unpaid ‘invisible’ work/ML (0–100)	62.78	27.75	0–100	
perceived burden of unpaid ‘visible’ work (0–100)	57.15	24.43	0–100	
PANAS positive (5–25)	13.76	3.79		
PANAS negative (5–25)	10.26	3.62		
GAD-7 (0–21)	7.63	4.75		
PHQ-2 (0–6)	2.07	1.49		

Notes. PANAS = Positive and Negative Affect Schedule, GAD-7 = scale for Generalized Anxiety Disorder, PHQ-2 = Patient Health Questionnaire for depressive symptoms, ML = Mental Load.

**Fig 1:**
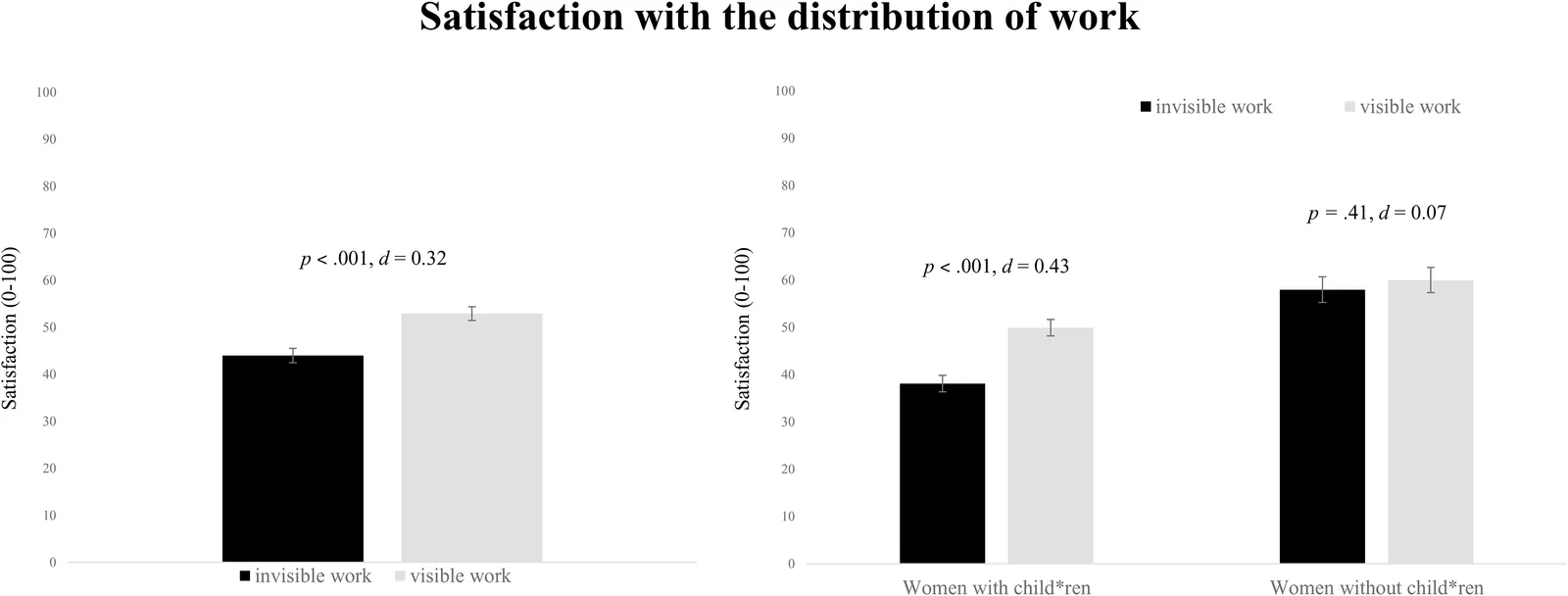
Satisfaction with the distribution of invisible and visible work. Left: divided into invisible and visible work; right: additionally, into women with and without children.

**Fig 2:**
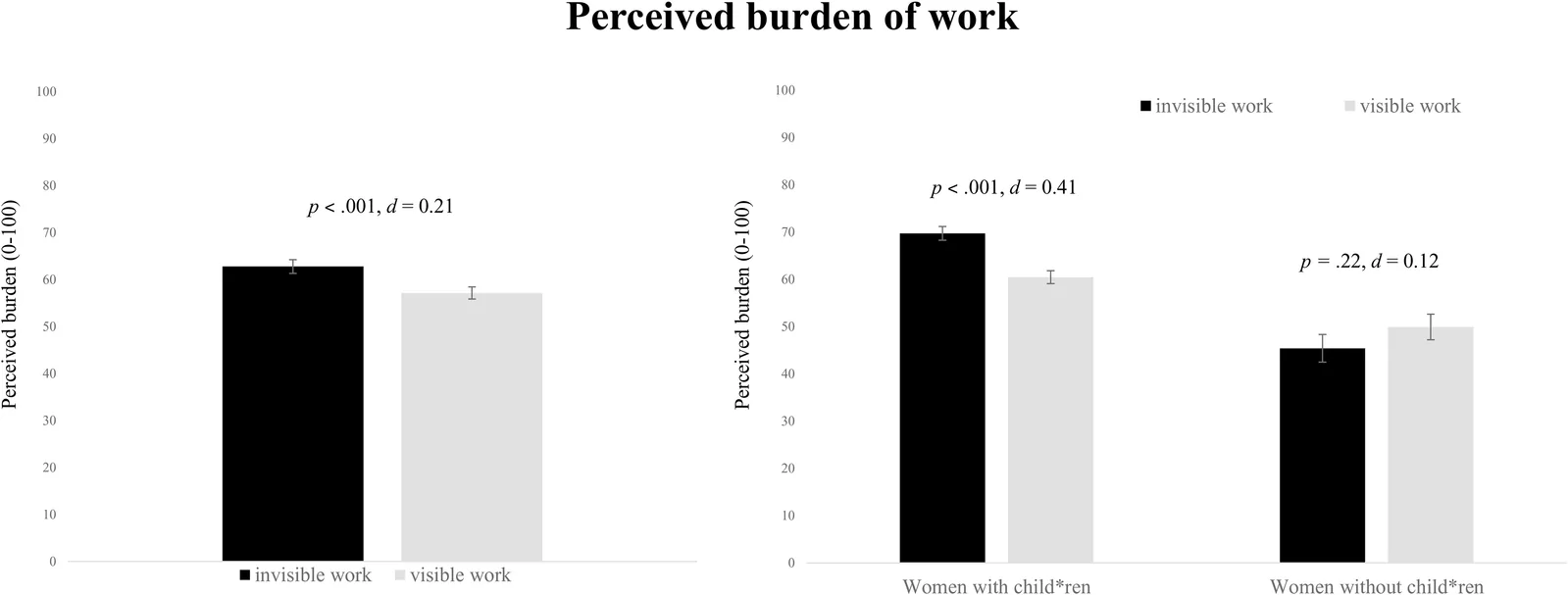
Perceived burden of invisible and visible work. Left: divided into invisible and visible work; right: additionally, into women with and without children.

The women are significantly more dissatisfied with the distribution of the invisible compared to the visible household-/care work (*t*(353) = 6.53, *p* <.001, *d* = 0.32). Here, marked differences were found between women with and without children (*F*(1, 352)=11.03, *p* ≤ .001, η^2^ = .03), with women with children being particularly dissatisfied with the distribution (*d* = 0.43) compared to women without children with no significant difference between invisible and visible work (*d* = 0.07); see Fig 1). Invisible household tasks are experienced as significantly more burdensome than visible tasks, but only with a small effect (*t*(367) = 3.79, *p* <.001, *d* = 0.21). When looking separately at women with vs. without children, it is noticeable that women with children experience invisible tasks as more stressful (*F*(1, 366)=15.75, *p* ≤ .001, η^2^ = .03), Fig 2).

#### Reasons for mental load.

Table 2 shows percentages for the reasons given for the burden of invisible tasks (multiple answers possible; study 1 and 2 comparative).

**Table 2:** Reported reasons areas of burden of invisible mental labor in household/care work in Study 1 (*N* = 368 women) and 2 (*N* = 1871 mothers).

Reasons for burden	*Study 1*	*Study 2*
**Invisible tasks**		
Constant thoughts about it	67.7%	92.8%
Tasks are never finished	59.5%	81.4%
Amount/frequency	52.2%	–
Reminding/explaining/discussing others	51.4%	–
(Sole) Responsibility	51.1%	48.5%
Low/lack of appreciation	50.5%	64.4%
No breaks/no time for oneself	47.8%	66.9%
Unequal distribution between partners	39.9%	51.1%
Perfectionism/ pressure to perform	35.1%	30.0%
No way to break out of social role	27.4%	54.1%
Multitasking	29.9%	64.8%
Complexity/intensity	25.8%	–
Low self-efficacy/outcomes not tangible	25.3%	–
Lack of balance	–	59.0%
Lack of payment	18.5%	45.6%
Find nothing stressful about it	2.4%	0.2%
Other (e.g., boredom, monotony, partners motivation)	2.2%	1.9%
**Superordinate areas of burden**		
Coordination of appointments	–	67.7%
Household	–	66.1%
Errands	–	67.9%
Children	–	85.2%
Other areas		4.6%

Notes. The fields left blank were not collected in the respective study. Responses entered in the “Other” category were summarized into overarching categories where appropriate.

Reasons given for the burden of visible tasks were (multiple answers possible): 66.8% never finished/repeated, 62.2% amount/frequency, 45.9% no breaks/time for oneself, 45.7% low/lack of appreciation, 39.1% conflict between own needs and those of others, 35.5% unequal distribution between partners, 28.5% perfectionism, 26.1% intensity/physical demands, 23.9% multitasking, 23.4% (sole) responsibility, 23.1% no possibility to break out of social role, 22.8% constant reminding/explaining/discussing, 22% lack of payment, 16.8% low self-efficacy expectation/results little visible, 2.4% find nothing stressful about it and 2.7% other (e.g., boredom, monotony, partner’s motivation).

#### Relationships between mental load and affect, anxiety, and depressive symptoms.

Mental load, i.e., perceived burden of invisible work, showed a significant negative correlation with positive affect (*r*_*s*_ = −.13, *p* = .011) and significant positive correlations with negative affect (*r*_*s*_ = .36, *p* <.001), generalized anxiety (*r*_*s*_ = .33, *p* <.001), and depressive symptoms (*r*_*s*_ = .26, *p* <.001).

## Study 2

Study 2 aims to expand the investigation of risk factors for mental load. In study 1, it was found that women with children (vs. without children) are dissatisfied with the distribution of invisible tasks in the family context and suffer from them to a significantly greater extent. In addition, the role of personality traits will be examined and whether the exact status of mothers (single parents) plays a moderating role.

### Method

#### Participants.

Potential participants for the Online survey ‘Mental load in mothers’ were recruited (conducted between December 13, 2022, and January 3, 2023) via university email distribution lists and, in particular, diverse social media platforms (e.g., Instagram, X) to enhance sample heterogeneity. The study addressed individuals who were at least 18 years old, considered themselves to be of the female gender (see title ‘mothers’, with diverse/non-binary not excluded), and living with at least one child in a shared household. Completion of the online survey took approximately 15 minutes. No raffle or other incentive was offered; however, psychology students could receive 0.5 course credits for participation where applicable. A required sample size of minimum *N* = 73 was calculated a priori (G* Power 3.1.9.7 [31]: linear multiple regression (two tails) f ^2^ = 0.15, α = 0.05, 1-β = 0.90). *N* = 1872 mothers took part in the survey. One person reported an age of ‘9’ for (her)self; because this contradicted the inclusion criterion (regardless of the reason, e.g., typing error), this person was excluded, and the final sample included *N* = 1871 mothers (99.9% female, 0.1% diverse/non-binary; *M* = 38.18, *SD* = 5.18 years, range 24–61 years). Women reported to have an average of *M* = 1.86 (*SD* = 0.73) children, ranging in age from 1 month to 34 years (*M* = 6.18, *SD* = 4.39 years). 75.4% of the women were married, 18.1% live in a relationship, 4.2 reports to be separated/divorced, 2.4% currently no partnership. 7.6% (*N* = 143) are single mothers. 74.3% have a university degree, 19.4% have a high school degree, 5.2% secondary school degree, and 1.0% other. In terms of employment status, 63.0% are working part-time, 17.0% are working full-time, 3.2% are students or in training, and 14.9% are currently unemployed (also housewife, parental leave, pension etc.). The ethics committee of the Psychological Institute of the Johannes Gutenberg-University Mainz has approved the study project. All participants provided written informed consent prior to participation, including consent for the scientific use of their anonymized data. Data were processed in accordance with applicable data protection regulations.

#### Measures.

Two questions were asked to capture mental load: Frequency and strength of the experienced burden of invisible daily tasks in the context of household/care work (7-point Likert scale for strength and frequency: 1 = not at all burdened, 2 = slightly burdened, 3 = rather slightly burdened, 4 = moderately burdened, 5 = rather burdened, 6 = burdened, 7 = strongly burdened; 1 = never, 2 = rarely, 3 = rather rarely, 4 = sometimes, 5 = rather frequently, 6 = frequently, 7 = very frequently). An average of the two questions was taken. The items showed an internal consistency of α = .84. Analogous to Study 1, possible reasons for the burden were asked in MC format (multiple responses possible, see Table 2). In addition, the survey asked in which of the areas ‘children’, ‘household’, ‘any appointment coordination’, ‘errands’ and/or ‘other things’ the mental load is perceived most strongly (multiple answers possible).

The personality traits of interest, neuroticism and conscientiousness have been screened with the short version of the Big Five Inventory (BFI-S [38]). Each dimension consists of 4 items which can be answered using a 5-point Likert scale (1 = disagree strongly, 2 = disagree a little, 3=neither agree nor disagree, 4 = agree a little, 5 = agree strongly). In this study, the BFI-S showed acceptable internal consistencies (α = .80 for neuroticism and α = .71 for conscientiousness).

#### Statistical analyses.

For all analyses, SPSS Statistics 23 [35] and the PROCESS macro for SPSS [39] (model 1) were used. Normality of the study variables was assessed using the Shapiro–Wilk test. As several variables significantly deviated from a normal distribution, Spearman rank correlations were used to examine bivariate associations. Moderation analyses were conducted using the PROCESS macro (Model 1) with 5,000 bootstrap samples to estimate bias-corrected 95% confidence intervals. To minimize potential sources of bias, no missing values were permitted in the dataset, as the online survey required all items to be completed before submission. This ensured complete data for all variables without the need for imputation. The statistical analysis plan, including the alpha level for significance testing (p < .05), was determined prior to data analysis.

### Results

#### Reasons and areas for burden.

Table 2 shows percentages for the reasons and areas given for the burden of invisible tasks (multiple answers possible; study 1 and 2 comparative). The most frequently mentioned reason of stress is the continuous thinking about everyday tasks and responsibilities (92.8% of mothers). The main area that is perceived as the most stressful is tasks related to care work for children (85.2%).

Further descriptive statistics for Study 2 are shown in Table 3. Overall, mothers reported high levels of mental load (M = 11.63, SD = 1.81; possible range 2–14). Mean conscientiousness was 3.93 (SD = 0.63) and mean neuroticism was 3.30 (SD = 0.85; both on a 1–5 scale).

**Table 3:** Means (*M*) and standard deviations (*SD*) of the used questionnaires.

Variables	M	SD
Mental Load (2–14)	11.63	1.81
Conscientiousness (1–5)	3.93	0.63
Neuroticism (1–5)	3.30	0.85

#### Relationships between mental load and conscientiousness and neuroticism.

Mental Load correlated weakly positively with conscientiousness (*r* = .14, *p* < .001) and moderately positively with neuroticism (*r* = .30, *p* < .001).

#### Single parent as moderator.

**Fig 3:**
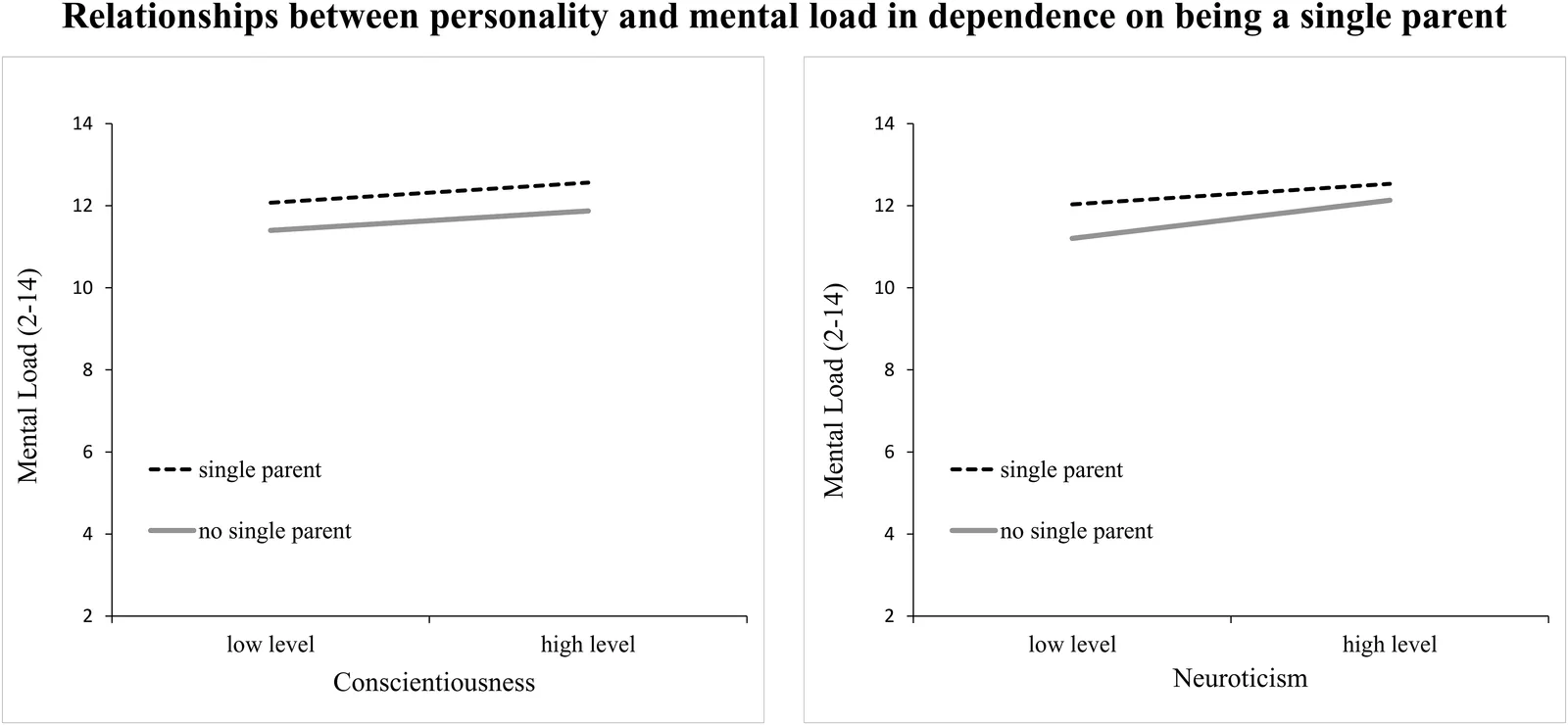
Single parent status as moderator for the relation between personality traits and mental load. Left: for the personality trait conscientiousness; right: for the personality trait neuroticism.

Being a single parent (single parent = 1, no single parent = 2) was not a significant moderator of the relationship between conscientiousness and mental load, b = −.39, *p* = 0.103, ΔR^2^ = 0.14%, *F*(1, 1867) = 2.66, *p* = .103. In contrast, single parent significantly moderated the link between neuroticism and mental load, B = .49, *p* = 0.014, ΔR^2^ = 0.29% *F*(1, 1867) = 6.06, *p* = .0139. The overall model was significant, *F*(3, 1867) = 73.32, *p* < .001, with variance clarification of 10.54%. In contrast to the single mothers (*r* = .09, *p* = .289), only the non-single mothers showed a significant correlation between neuroticism and mental load (*r* = .31, *p* <.001). Fig 3 shows the moderation analyses.

## Discussion

As mental load in the context of home/care work has been comparatively little studied so far, two studies aimed to investigate causes of mental load and associations with personality traits and psychological symptoms. Across two studies, we found that mothers experienced invisible domestic/care tasks as particularly burdensome and unequally distributed. Regarding hypothesis testing, H1 and H2 were supported (i.e., invisible tasks were rated as more burdensome than visible tasks, and mothers reported greater perceived inequality/imbalance, with effects especially pronounced among mothers). H3–H5 were supported (i.e., higher mental load was associated with higher symptoms of anxiety and depression and with higher neuroticism). H6 was not supported (conscientiousness was not negatively related to mental load but showed a small positive association). Finally, H7 was partially supported (personality moderated the mental load–symptom association for neuroticism but not for conscientiousness). In the following, we examine these findings in more detail and discuss them in light of prior literature, implications, and limitations.

### Burden of invisible vs. visible daily tasks and causes of mental load

Study 1 showed that invisible household/care work such as planning and organizing was perceived as significantly more unequally distributed and significantly more burdensome than visible tasks, with these significant differences attributable to women with children (women without children reported no differences between invisible vs. visible tasks). This means that Hypothesis 1 was supported for mothers, but not for women in general. It confirms that mothers actually experience processes such as planning, organizing, relating, and emotional work as more stressful compared to visible daily tasks such as cooking and washing. These findings are consistent with the conclusions of the systematic review by Reich-Stiebert et al. [16] and extend beyond the studies summarized therein by providing a direct comparison between ‘visible’ and ‘invisible’ tasks. There are various reasons why there are marked differences between women with and without children. For parents, the birth of children increases the quantity, complexity and, in particular, the emotional component (e.g., associated with worrying about the child, calming the children) of invisible tasks [3], which can be experienced as stressful. The component of boundless invisible tasks mentioned by Dean et al. [3] also increases (e.g., taking care of children around the clock). In particular, family formation/birth of a child may be associated with a traditional distribution of social roles in heterosexual relationships in Western countries (either the more traditional distribution returns if it was previously egalitarian or it consolidates) [40,41]. Social norms and high expectations in the sense of ‘idealized motherhood’ (on an individual and societal level) may also play a role in mothers [42,43]. This is also confirmed by a recent literature review [16], which summarized that women take on more mental labor, especially in the areas of children and parenting, and that this unequal distribution brings more negative effects, such as a higher family-to-work spillover [16,44].

These findings are consistent with the Transactional Stress Model [12] outlined in the theoretical section, as the reported burden of mental load varied substantially between individuals. Some participants indicated no burden at all, suggesting that the subjective appraisal of cognitive, invisible task (e.g., rather than their objective quantity) may be the more critical factor in determining perceived strain.

Consistent in Study 1 and Study 2, the most frequently reported cause (68/93%) of mental load was constantly thinking about everyday tasks, followed by tasks never being finished (60/81%). This also confirms the assumption of Dean et al. [3] that the characteristics of invisible tasks of being ‘limitless’ and ‘continuous’ in particular account for the load. The third characteristic of Dean et al. [3], the invisible (not perceiving or not acknowledging) was given by about half of the women and 2/3 of the mothers as a cause of stress. The fact that Study 2 found higher scores overall is probably because Study 2 surveyed mothers only and Study 1 surveyed women in general (see burden on mothers also previous section).

This is also in line with the finding from Study 2 that mothers reported caring for children as the most stressful area (compare also above-mentioned review by [16]).

### Mental load and psychological symptoms

Studies on the relationship between mental load and affect and psychopathology are currently still scarce, with most studies focusing on parental burnout [e.g., 14,20]. Our study confirmed the findings by Offer [6] that mental load is negatively related to positive affect and positively related to negative affect in women. The effect size was significantly larger for the negative effect, indicating that mental load is more likely to be related to increased negative affect, rather than reduced positive affect.

We derived the hypothesized associations with generalized anxiety and depressive symptoms from studies of the association of family effort (vs. reward) and stress in mothers during the COVID-10 pandemic and anxiety and depressive symptoms [10,11,25]. We have now also confirmed that mental load is also positively related to generalized anxiety and depressive symptoms, which confirmed our hypotheses 3 and 4. In an earlier study [25], it was shown that mental load is associated with feelings of being overwhelmed and overburdened, which may be related to or contribute to anxiety. The results of the present studies show this specifically for generalized anxiety. Regarding depressive symptoms, a recent study [24] also found that the distribution of cognitive domestic labor is related to depressive symptoms. Complementing this, our study showed that the perceived burden of unpaid/invisible household tasks is related to depressive symptoms. This study does not allow any causal conclusions, both mental load and anxiety or depression could increase the vulnerability of the other factor and vice versa, which could also result in a vicious circle. Our findings are consistent with the study by Kaplan [45], who found that, in housewives, co-dependence (the tendency to prioritize the needs of others over one’s own) was associated with psychological symptoms such as anxiety and depression. Furthermore, it clarifies or expands on another possible toxic feature of mental load, which was mentioned above as including responsibility, emotional labor, and relationship work.

### Personality traits and moderation

Based on previous stress and burnout research, a negative relationship between conscientiousness and mental load and a positive relationship between neuroticism and mental load were postulated [20,32,33]. We were able to confirm the negative link between neuroticism and mental load with a moderately strong effect (hypothesis 5). Individuals with higher neuroticism tend to be more sensitive to stressful situations and experience slower recovery [46], which was supported by the study of Suls and Martin [47] showing higher physiological reactivity and subjective perception of stress in individuals with higher neuroticism. In stressful situations, neuroticism also appears to be related to lower resilience [48]. The result on neuroticism also confirms the finding from Study 1 on negative affect, as both constructs overlap considerably. Taken together with our finding, neuroticism seems to make people more vulnerable to stress in the context of non-visible daily household/care tasks.

Regarding conscientiousness, we found a positive relationship with mental load, with a very small effect, which contradicts our hypothesis 6. Le Vigouroux et al. [27] found that low conscientiousness was associated with parental burnout (i.e., a negative association). While we referred to previous stress research (such as the study by Le Vigouroux [27]), it is conceivable that differences exist here between stress/burnout and mental load in that conscientiousness (e.g., doing tasks thoroughly, making plans) in the context of care/domestic work is more likely to be related to more mental load in the sense of a tendency to do tasks very thoroughly or accurately. For example, previous studies showed that perfectionism and the desire/pressure to be an ideal mother are associated with increased stress [42,43]. In our studies, about one-third of women and mothers reported perfectionism or pressure to perform as a cause of mental load.

Finally, we found that being a single mother moderates the relationship between neuroticism and mental load, in that the relationship is significant only for non-single mothers (hypothesis 7). Looking at Fig 3, mental load appears to be consistently elevated in single mothers largely independent of neuroticism. In contrast, for non-single mothers, a higher mental load is found for individuals who have higher levels of neuroticism, i.e., for non-single mothers, neuroticism seems to play a larger role on mental load. As discussed above, there is higher stress reactivity and lower stress resilience associated with neuroticism, which could make individuals more vulnerable to stress from unseen everyday tasks [46–48].

With regard to conscientiousness, the moderation analysis was not significant. Fig 3 shows that the correlation between conscientiousness and mental load is very similar regardless of whether a person is a single parent. It is possible that the correlation between the personality trait of being very careful, disciplined, and good at time management and the burden of invisible tasks is independent of relationship status. Conscientiousness also appears to be more strongly related to relationship status. For example, Stern et al. [49] found that long-term singles have lower scores in conscientiousness.

### Practical implications

Based on the reported burden or load of invisible house/care work, particularly in mothers, the reported causes, and associations with neuroticism and psychopathology, implications arise at the individual, couple, and societal levels. These are likely to be far-reaching, but we limit ourselves here to these with more specific reference to the reported findings.

Also, as Dean et al. [3] similarly pointed out, reduction of mental load could be promoted by making tasks visible (e.g., couple level), as well as improved conditions regarding boundarylessness and chronicity, e.g., via improved support networks or more egalitarian distribution. Mothers in particular perceived an uneven distribution and mental load, with about one-third citing perfectionism as the cause of mental load and about half citing not being able to break out of social roles. Although we did not explicitly ask this, the issues of traditional social roles and the perfect mother as an ideal are worthy of social scrutiny, as they also contribute significantly to gender inequality and strain [42,43]. This also leads to future research ideas that would be important to advance such as studying same-sex couples and longitudinal studies such as how mental load changes before and after the birth of a child. Also, since mental load can have an unfavorable impact on mental health and psychopathology, it is crucial to reduce mental load through, for example, the above-mentioned interventions. Given that mental load and psychopathology presumably interact in a kind of vicious circle, it is also important to recognize psychological symptoms at an early stage, to provide support services (e.g., expansion of low-threshold counseling and prevention), and, if a mental disorder is present, to keep the issue of mental load in mind during psychotherapy (since mental load could also be a perpetuating factor in psychopathology). Since (social) gender plays a crucial role in determining the living environment and life tasks (such as the take-over of care work) in many cultures, it is important to keep these aspects in mind when considering interventions or therapies. For example, Kaplan [50] suggests a feminist therapy that specifically addresses the needs of women (e.g., feelings of guilt).

### Limitations and future research

The women in study 1 and 2 had a higher level of education compared to the general German population, so the study is not representative in this respect. For example, it was found that in partnerships in which the woman earns more, the men take on proportionately more domestic work (unequal distribution lower) compared to men in which women tend to earn the same/less [51]. Although participation in both studies cannot be completely ruled out, the studies were conducted independently and used different recruitment emphases, particularly with a stronger focus on social media in Study 2. Therefore, substantial overlap between the samples is considered unlikely. Our study has a specific focus on mental load, personality traits and psychological symptoms in women and mothers. In terms of gender, the studies are one-sided (social role as a woman) and cannot be generalized to men (social role/affiliated)/non-binary/diverse. Even though Kaplan [11,45], for example, found similar results for Turkey, it would be very interesting to address the question of mental load in the context of the family across cultures or in a more heterogeneous way. Furthermore, only a relatively small proportion of participants in Study 2 were single mothers (7.6%). Consequently, moderation analyses involving single-parent status should be interpreted with caution, as the limited subgroup size may have reduced statistical power and the stability of the corresponding estimates. Overall, it would be helpful for the generalization of the results if the samples had been more heterogeneous and representative in terms of sociodemographic variables (gender/affiliation, age/children’s age, education). Future studies should also consider additional family characteristics, such as the number of children and whether a child has special needs, as these factors may further contribute to differences in maternal mental load.

In addition, many other potential risk factors were not examined, such as previous psychiatric illnesses, relationship quality/couple conflicts, physical illnesses of family members, which should be considered in further studies. With regard to the assessment of the construct mental load, no validated questionnaire is available yet, so that the comparability with other studies is limited. Thus, it is highly relevant for future research to develop and validate an appropriate questionnaire. Because of the cross-sectional design, our study does not allow us to draw causal conclusions. As mentioned above, future longitudinal studies would be promising to examine changes over time (e.g., before and after childbirth) and to identify predisposing factors and consequences of mental load.

## Integrated summary and conclusion

The two present studies with women and mothers are, to our knowledge, the first to systematically compare the perceived burden of invisible care/household tasks (mental load) with that of visible tasks, to identify causes of mental load, and to examine its associations with personality traits and psychopathology. Consistent with previous findings [5,16], our results show that mothers perceive invisible everyday tasks—such as planning, organizing, and emotional management—as more burdensome and less equally distributed than visible tasks (e.g., cooking, laundry), largely because they are experienced as ongoing and limitless [52].

Building on the Transactional Stress Model [12], mental load was operationalized as the subjective burden of invisible household and care tasks, thereby extending previous research by incorporating additional predictors and their interactions. Mental load was positively associated with negative affect, neuroticism, and generalized anxiety. Notably, among mothers in partnerships, mental load increased with higher levels of neuroticism, whereas this pattern was not observed for single mothers.

With regard to affective and psychopathological symptoms, our results extend prior burnout and stress research [14,17,20,53] specifically to the domain of mental load. Finally, we highlight implications at multiple levels: individual (reducing vulnerability, strengthening resilience, low-threshold support), dyadic (more equitable task distribution, addressing social gender norms), and societal (structural improvements in care infrastructure, prevention and intervention measures, broader gender norm change).
