# Supplementary material for: Mental load in women and mothers: Causes of distress, personality traits, and psychopathology
Source: PLoS One. 2026 Sep 2;21(9):e0356255. doi: 10.1371/journal.pone.0356255 (PMC13537576; doi:10.1371/journal.pone.0356255)
Supplement: Supplementary file 1 [file pone.0356255.s002.docx]

Dear Dr. Ioana Gutu,

dear reviewers,

Thank you very much for your letter, and for the very helpful reviews of our manuscript titled *‘Mental load in women and mothers: Causes of distress, personality traits, and psychopathology’* (PONE-D-23-25403).

We thank you and the reviewer for the thoughtful and constructive feedback and suggestions for improving the manuscript. We have carefully revised the manuscript as recommended (one version with tracked changes) and address the comments point by point below.

**Comments editor:**

**1.) Please ensure that your manuscript meets PLOS ONE's style requirements, including those for file naming. The PLOS ONE style templates can be found at**

**https://journals.plos.org/plosone/s/file?id=wjVg/PLOSOne_formatting_sample_main_body.pdf and**

**https://journals.plos.org/plosone/s/file?id=ba62/PLOSOne_formatting_sample_title_authors_affiliations.pdf**

[AU]: In the revised version, we have checked and revised the style requirements, such as levels and formatting of headings and figure citations/file naming, and we have deleted information of the corresponding author (only E-Mail).

**2) Please include your full ethics statement in the ‘Methods’ section of your manuscript file. In your statement, please include the full name of the IRB or ethics committee who approved or waived your study, as well as whether or not you obtained informed written or verbal consent. If consent was waived for your study, please include this information in your statement as well.**

[AU]: Thank you for the helpful comment. We have inserted additional information in the Method section.

*‘The ethics committee of the Psychological Institute of the Johannes Gutenberg-University Mainz has approved the study project.’* (on pages 8 and 15)

On pages 7 (Study 1) and 14 (Study 2) in the first submission (and now the revision), we described the written informed consent (‘*After providing written informed consent…, ‘Written informed consent was required prior to participation.*’).

**3) Please include a separate caption for each figure in your manuscript.**

[AU]: Thank you for this hint. As recommended, we have inserted captions for each figure (see pages 11, 12 and 16).

**Comments reviewer**

**Reviewer #1:**

1. **What are the main claims of the paper and how significant are they for the discipline? The paper focuses on mental load in mothers to better explain the mechanisms underying mental health suffering in this population through two studies : 1) the first study to analyse visible and invisible tasks that contribute to mental load and the relationship with mental health; 2) the second study to analyse the burden of invisible daily tasks in the context of household/care work.**

**2.Are the claims properly placed in the context of the previous literature? Have the authors treated the literature fairly?**

**The thematic of mental burden of mothers is properly placed in the context of the previous literature but the authors recongnize that previous data on this topic are scarce.**

**The aims are not enough clearly described.**

**Hypothesis are not enough justified and argued and that consititute a big methodological issue: why chosing only women in the study to consider mental burden and why exlude men ? Why do the authors do not consider other important factors that coould contribut eto mental burden such as psychiatric antecedents, traumatic events, conflict couples, children illness.... There is to much "a priori" hypothsesis" and perhaps that a qualitative study protocol could have helped to better investigate it.**

[AU]: Thank you for the positive feedback mentioned in the introduction. As recommended, we have specified the objectives in the revised version and derived hypotheses that are theoretically better founded. We integrated theories and derived our hypotheses from them. In addition, we conducted a new literature search (e.g., meta-analysis by Ren et al., 2024). We can fully understand your questions, would like to explain them here and have expanded the discussion and limitations in this regard. The study objectives were a compromise between building on the previous ones (especially the target group) and expanding the previous findings (e.g,. to specify mental load and mental health in general for women here or to derive burnout research from the area of mental load). We fully agree that this should be described in the manuscript for a better understanding.

*“These characteristics are supported by the Transactional Stress Model [13], which states that the subjective evaluation of a situation/event as stressful is crucial, as are the individual resources available to cope with the situation.”* (On page 5)

*“A recent systematic review [18] identified 26 articles on parental burnout and categorized associated factors according to the Ecological Systems Theory (EST) [19]. In particular, factors at the individual microsystem level, e.g., sociodemographic data, personality factors, and anxiety/depression, were found to be associated with parental burnout. The latter (mental health problems) e.g., due to the overlap of dysfunction of the hypothalamic-pituitary-adrenal axis (stress axis). The (risk) factors associated with parental burnout may resemble the mental workload, although there is a lack of systematic scientific research into the reasons why mental labor is perceived as stressful and how mental load is related to mental health.”* (on pages 5 and 6)

*“In this paper, we aim to fill these research gaps with two studies (risk factors of mental load, associations of mental load with personality traits and mental health symptoms).”* (on page 6)

*“Given the characteristics of mental load and the and the overlap with parental burnout, we assumed that the ‘invisible’ tasks are perceived as more burdensome than the ‘visible’ ones and that the perceived burden of ‘invisible’ tasks is positively related to anxiety and depressive symptoms.”* (on pages 5 and 7)

*“In the context of parental burnout, there is also the assumption that neuroticism is associated with deficits in emotion regulation and that emotionally unstable parents react more strongly to life events, which can increase the likelihood of parental burnout [18]. So similar to findings in burnout and stress research [18, 21, 25, 26], it could also be hypothesized that neuroticism is positively related to mental load.”* (on page 7)

*“Our study has a specific focus on mental load, personality traits and psychological symptoms in women and mothers. In terms of gender, the studies are one-sided (social role as a woman) and cannot be generalized to men (social role/affiliated)/non-binary/diverse. In addition, many other potential risk factors were not examined, such as previous psychiatric illnesses, relationship quality/couple conflicts, physical illnesses of family members, which should be considered in further studies.”* (on page 22)

**3. Do the data and analyses fully support the claims? If not, what other evidence is required?**

**PLOS ONE encourages authors to publish detailed protocols and algorithms as supporting information online. Do any particular methods used in the manuscript warrant such treatment? If a protocol is already provided, for example for a randomized controlled trial, are there any important deviations from it? If so, have the authors explained adequately why the deviations occurred?If the paper is considered unsuitable for publication in its present form, does the study itself show sufficient potential that the authors should be encouraged to resubmit a revised version?**

**Despite this important topic, there is too much methodological issues to consider this**

**paper for a publication.**

[AU]: Thank you for your feedback. The aim of our studies was to advance the new research field of mental load and, based on our results, to encourage further research in this area. In our limitations, we discuss weaknesses of the studies, and as recommended, we have expanded them.

**4.If the paper is considered unsuitable for publication in its present form, does the study itself show sufficient potential that the authors should be encouraged to resubmit a revised version?**

**No; 5. Are original data deposited in appropriate repositories and accession/version numbers provided for genes, proteins, mutants, diseases, etc.?**

**Yes, the details of the methodology are sufficient to allow the experiments to be reproduced? ; 6.Is any software created by the authors freely available?**

**Yes, there is a software created by the authors freely available.**

**7. Is the manuscript well organized and written clearly enough to be accessible to non-specialists?**

**No, the manuscript is not well organized and written clearly enough to be accessible to non-specialists, because for example they do not explain why neuroticism and perfectionnism could be in link with mental burden.**

[AU]: We would like to thank the reviewer for suggesting that we explain the hypotheses regarding the connection between personality traits (neuroticism and conscientiousness) and mental load in more detail. We have made additions to the revised version (see also point 1).

*“Study 2 addressed the relationship between the personality traits neuroticism as well as conscientiousness and mental labor in the context of family. Individuals with higher neuroticism are considered to be more emotionally unstable, sensitive, and tend to become more easily unbalanced under stress. In the context of parental burnout, there is also the assumption that neuroticism is associated with deficits in emotion regulation and that emotionally unstable parents react more strongly to life events, which can increase the likelihood of parental burnout [18]., Sso similar to findings in burnout and stress research [18, 21, 25, 26], it could also be hypothesized that neuroticism is positively related to mental load. Regarding conscientiousness, individuals with higher levels are thought to be more organized, disciplined, and have better time management [21], and negative associations have been found between conscientiousness and parental and job burnout [21, 25]. Accordingly, we assume a negative correlation between conscientiousness and mental load in the context of family.”* (on page 7)

**8.Is it your opinion that this manuscript contains an NIH-defined experiment of Dual Use concern?**

**No**

**Reviewer #2:**

**-It may be a good idea to write the title "Introduction" when starting the introduction section.**

[Au]: Thank you very much, we have added this heading.

**-In the introduction section, more current literature can be shared about how women are affected by stressful life events and what consequences this effect has.**

**Data can be shared about the psychological symptoms experienced by women, especially during and after the pandemic.**

**The study https://doi.org/10.35365/ctjpp.21.2.13 can be used.**

[Au]: We thank you very much for the recommendation of this very interesting article, which we were pleased to expand. In addition, we had carried out a literature search and added current literature (e.g. Ren et al., 2004, systematic review on parental burnout).

*“During the COVID-19 pandemic (particularly during the lockdown), much of the double burden (housework, care work including homeschooling, and paid work, respectively) fell on women and mothers, who reported increased levels of burnout, loneliness, anxiety and depression during this time [12, 13].”* (on page 4)

**-The results section has very understandable and impressive data.**

[Au]: Thank you for the positive feedback. We are very happy to hear that.

**-I believe that it would be good to compare your target group with other similar studies in the discussion section.**

**You can benefit from the study** [**https://doi.org/10.1007/s11469-022-00910-1**](https://doi.org/10.1007/s11469-022-00910-1)**.**

[Au]: Thank you. We absolutely agree that this is a very interesting addition to our discussion:

*“Our findings are consistent with the study by Kaplan [41], who found that, in housewives, co-dependence (the tendency to prioritize the needs of others over one's own) was associated with psychological symptoms such as anxiety and depression. Furthermore, it clarifies or expands on another possible toxic feature of mental load, which was mentioned above as including responsibility, emotional labor, and relationship work.”* (on page 20)

**- I think it is important to talk about the importance of working with gender-sensitive approaches in the detection and treatment of women's mental problems in the "Practical implications" section. this study "https://doi.org/10.35365/ctjpp.21.3.22" can be used.**

[AU]: We agree with you that this is a very important topic, which we have added to. In addition, based on the recommendation of another reviewer, we have also expanded the limitation.

*“Since (social) gender plays a crucial role in determining the living environment and life tasks (such as the take-over of care work) in many cultures, it is important to keep these aspects in mind when considering interventions or therapies. For example, Kaplan [45] suggests a feminist therapy that specifically addresses the needs of women (e.g,, feelings of guilt).”* (on page 22)

*“In terms of gender, the studies are one-sided (social role as a woman) and cannot be generalized to men (social role/affiliated)/non-binary/diverse.”* (on page 22)

**Reviewer #3: The need for a professional editor or a review from a native speaker was evident already in the abstract. For example, “also” in the first sentence of the abstract implies there was something before this that this is adding onto…many issues with the writing that could be pointed out by an editor.**

[AU]: Thank you for your extremely helpful feedback. We have reviewed the wording. We have deleted “also” in the abstract.

**Why include this parenthetical comment “(preceding, parallel, following)”—I found this made the sentence much less clear because of this..**

[Au]: We absolutely agree with you that the sentence is easier to understand without the brackets, and we have deleted them.

**Line 60—should be amount rather than extent**

[Au]: Thank you very much, we have exchanged words.

**I found the paragraph (lines 54-76) about different related concepts (cognitive labor, mental load) unnecessarily complex—why bring up unrelated concepts (e.g., “cognitive dimension”) rather than simply defining the words you would use--mental labor and mental load—and then using those words? Throughout the paper these selected terms should be used consistently (e.g., on line 95 mental labor is called cognitive work and on page 5 in general, or in the discussion sometimes the term “burden” is used in the place of load)**

[Au]: We thank the reviewer for the helpful recommendations. We have now simplified the introduction with the definitions and standardized the use of terms.

*Deleted: “According to Sweller et al. [6], cognitive load refers to the load on working memory, whereby the capacity for the amount of information is limited. Mental workload, as defined by Young and Stanon [7, p. 507], describes the extent of "attentional resources required to meet both objective and subjective performance criteria".* (on page 3)

*“Dean et al. [3] describe three characteristics, in particular, that contribute to the stressful and exhausting nature of mental laborcognitive work: 1. these tasks and duties are ‘invisible’ (e.g., little valued), 2. they are boundaryless (e.g., flowing or interfering with falling asleep, paid work, or leisure), 3. they are ongoing (e.g., few short- or longer-term breaks).”* (on page 5)

*Discussion, for example:*

*“Mothers in particular perceived an uneven distribution and mental load, with about one-third citing perfectionism as the cause of mental load and about half citing not being able to break out of social roles.”* (on page 21)

**Line 83—physical symptoms**

[Au]: Thank you for pointing this out; we have reworded it.

**Line 158—the G*power analysis selected should be better explained and justified—a G*power analysis for a two-tailed correlational analysis with the parameters described returns a different sample size, and there are a number of analyses conducted (paired samples t-test, independent samples t-tests, and correlations). A mixed ANOVA would be the correct analysis for the findings on the right side of Figure 1 and 2, rather than this mix of individual tests. The G*power analysis could be conducted for this within-between interaction. For the correlation, it should be a two-tailed test as there was a negative predicted relationship in Study 2 between conscientiousness and mental load but a positive correlation was reported. This same answer of N = 88 is given for Study 2 without justification (Line 275). For Study 2, G*power analysis should be conducted for a moderation analysis (a regression with an interaction term) as that is the analysis used.**

[Au]: We absolutely agree with you that more background information should be provided for the G-Power analyses. In study 1, for example, we had paired t-tests (N=36) and correlations (N=88), one-sided (directed hypotheses in Study 1). Since the analyses for correlations were higher, we had only reported these. We absolutely agree with you. For study 1, we added a mixed ANOVA and extended the GPower analysis accordingly, and for study 2, we added the moderation analysis to the GPower analysis.

*“Paired t-tests were calculated to examine differences in the satisfaction of distribution and load of ‘invisible’ versus ‘visible’ work and a mixed ANOVA to additionally examine the effect of children (mothers with vs. without children).”* On page 10

*“A required sample size of at least N = 88 was calculated a priori [G* Power 3.1.9.7 [28] correlation, one tail: ρ = 0.3, α = 0.05, 1-β = 0.90; mixed ANOVA N = 62, f= 0.25, α = 0.05, 1-β = 0.90, correlation among measures=0.3”* (on page 8)

***“****A required sample size of minimum N = 73 was calculated a priori [G* Power 3.1.9.7 [28]: linear multiple regression (two tails) f ^2^ = 0.15, α = 0.05, 1-β = 0.90].”* (on page 15)

**Line 272 “see title ‘mothers’, with divers not excluded”—typo? Unsure what this sentence means.**

[Au]: Thank you for pointing out the typo. We meant the word “diverse”/non-binary (gender) and have corrected it.

**In Study 1, the major take-away that I think we are supposed to observe is that mothers are particularly dissatisfied with the distribution of invisible labor (which should be tested in one test, not through multiple separate independent and paired t-tests), and their perceived mental load from this is correlated with their well-being. It would be helpful to have a paragraph that summarizes this and provides a transition to Study 2. It might be helpful to have a visual model demonstrating how all of these different factors from the two studies are related (personality factors, family variables, mental load, well-being measures). This organizing framework (along with the predicted relationships) could have been pre-registered, and that would help with the haphazard feeling of the paper, along with a transition between Study 1 and Study 2. What do the authors think are the important findings of Study 1, and how is Study 2 related to these findings? The analysis for Study 2 focused only on mothers (which makes sense from the findings of Study 1) and their relationship status (but this is not introduced in the introduction to the study—there is only a single sentence introducing the idea that single mothers would be studied in Study 2 in the intro before Study 1). From the graph, it appears single mothers would report higher mental load, but the statistics are not reported for this. Is this significant? In the discussion it is reported as an effect (ll 403-404), but the statistics are not reported in the results section to know if this is actually a significant finding.**

[Au]: Thank you for your very helpful comments. As recommended, we added a mixed ANOVA for study 1 (see point above). We agree that the results of studies 1 and 2 should be discussed more comprehensively and have added this to the revised version. In addition, we have added the statistic that single mothers report a higher level of stress. E.g.,

*“Study 2 aims to expand the investigation of risk factors for mental load. In study 1, it was found that women with children (vs. without children) are dissatisfied with the distribution of invisible tasks in the family context and suffer from them to a significantly greater extent. In addition, the role of personality traits will be examined and whether the exact status of mothers (single parents) plays a moderating role.”* (on page 15)

**-- The children ranged in age up to 34 years old—it seems that the age range of children should be restricted to include mothers actively parenting their children as described in the discussion.**

**Discussion could be much stronger— both the organization and actual writing could be improved. Some sections seem more like a first draft (e.g., lines 415-432).**

**The following description of the significant finding (r = .14 , p < .001) is problematic: “Regarding conscientiousness, contrary to our hypothesis, we found a positive relationship with mental load, but with a very small effect (probably not significant in a smaller sample).” Yes, a correlation around r = .10 is considered a small effect size, but researchers cannot assert that a significant effect is not really significant (just as one can’t say an insignificant effect would be significant with a larger sample size).**

**Notes would be helpful for the figures (e.g., which study the data are from, what the error bars represent, if the low/high categories are a median split or +/-1 SD etc). In Figure 3, it would be helpful to use the term mothers rather than parents to be clear that this is only women.**

[Au]: Thank you for your helpful comments on how to improve the manuscript. We agree with you completely and have corrected the aspects you mention.

e.g. *“Regarding conscientiousness,, we found a positive relationship with mental load, with a very small effect.)* (on page 31)

**Reviewer #4: Thank you for writing such an interesting paper. It was a pleasure to read it. As a reader, it would be great to understand your sample's socio-cultural contexts to get a better perspective on their mental load. Since mothers are operating within the societal contexts and each context has its own boundaries, and implications on a mother's mental load, requesting authors to include this bit in the methodology as well as weave it in the discussion.**

[Au]: We agree with you that this is an important and interesting point, which we have added.

“Our study has a specific focus on mental load, personality traits and psychological symptoms in women and mothers. In terms of gender, the studies are one-sided (social role as a woman) and cannot be generalized to men (social role/affiliated)/non-binary/diverse. Even though Kaplan [12, 41], for example, found similar results for Turkey, it would be very interesting to address the question of mental load in the context of the family across cultures or in a more heterogeneous way.” (on page 23)

Yours sincerely,

The authors
