# Supplementary material for: Mental load in women and mothers: Causes of distress, personality traits, and psychopathology
Source: PLoS One. 2026 Sep 2;21(9):e0356255. doi: 10.1371/journal.pone.0356255 (PMC13537576; doi:10.1371/journal.pone.0356255)
Supplement: Supplementary file 2 [file pone.0356255.s003.docx]

Cover letter / Response to Reviewer #5

Dear Dr. Gutu,

Thank you very much for your careful re-review and for reiterating the points that still needed to be addressed. We appreciate the opportunity to improve the manuscript further. Importantly, we believe that one of the remaining issues, specifically the concern that the Introduction is too short, was due to a misunderstanding that unfortunately only became clear to us after receiving the present round of comments. In our previous revisions, we treated the full theoretical framing and conceptual development preceding the Methods as part of the Introduction/Background (i.e., everything above the Methods section). This interpretation is consistent with PLOS ONE’s general IMRAD expectations and the journal’s flexibility regarding the naming and ordering of middle sections. In addition, in preparing the revision we consulted recent PLOS ONE articles that use a concise introductory opening while integrating the broader conceptual framing within the pre-Methods section (e.g., Walsh et al., 2024; Shiely et al., 2024; Habibzadeh, 2024). Because our theoretical background (which we considered part of the Introduction) was already extensive, we initially avoided substantially lengthening the opening paragraph to prevent further manuscript expansion and to avoid redundancies.

We sincerely apologize for the confusion caused by our previous revisions. Based on both your earlier feedback and the current comments, we have revised the Introduction to make the manuscript structure explicit and easier to follow. Specifically, in the current revision we strengthened signposting at the start of the Introduction, added a brief roadmap outlining the sequence of the conceptual framing and the two studies, and ensured that the boundary between the Introduction/Background and the Methods section is unambiguous. We hope these changes resolve the misunderstanding and address your request while avoiding unnecessary duplication and further expansion of the manuscript.

Below we respond point-by-point to your comments. For transparency, we indicate where changes were made in the revised manuscript.

**1.** **Introduction length and structure:** Your Introduction has not been improved. Between all the versions, the current revision presents a manuscript with an Introduction shorter than the Abstract (copy of our Introduction). Perhaps it would be better if you re-organize the manuscript and write about the main chapters Introduction Lit Rev Methodology Conclusion Future research and Limitations. Please refer to the Introduction and revise it according to the previously submitted remarks. There are numerous parts missing from it.

Response: We clarified the manuscript structure so that it is unambiguous that the Introduction/Background comprises the full conceptual and theoretical section preceding the Methods (rather than only the opening paragraph). Specifically, we strengthened the introductory signposting and added a brief roadmap, while avoiding redundant expansion of points already covered in the theoretical background. We also aligned the overall organization with your suggestion by ensuring a clear progression from results to interpretation in light of prior literature, followed by practical implications, and ending with 'Limitations and Future Research'.

On page 3:

Introduction ‘…. *Beyond these observable activities lies a largely unseen layer of ongoing thinking work: remembering, anticipating, monitoring, coordinating, and emotion/relationship management. In everyday life, this ’mental load’ can be experienced as intrusive and difficult to switch off, because it is boundaryless and recurrent [3]. The invisible processes or cognitive work have received increasing attention in the media and society in recent years (e.g., during the COVID-19 pandemic), but are scientifically under-researched, including clear conceptualizations, correlates, and mechanisms [3–5]. To address these gaps, the present manuscript first differentiates key concepts (mental labor vs. mental load), summarizes existing evidence on mental health correlates and stressful characteristics, and then reports two empirical studies that (a) compare perceived burden of invisible vs. visible tasks, (b) identify the most frequently endorsed reasons for the burden, and (c) examine associations with psychological symptoms and personality traits. In the following Literature Review, we synthesize prior work on mental load and related concepts, with a focus on its stressful characteristics and associations with mental health and personality. We then derive the aims and hypotheses of the present research before detailing the Methods.’*

On page 4: *‘Literature Review’*

To enhance the clarity of the manuscript structure, we adjusted the formatting of the Literature Review subheadings by using a smaller font size.

On pages 28 and 29: As recommended, we have restructured the end of the manuscript: ‘*Conclusio’* after “*Practical Implication*’ and the manuscript ends with ‘*Limitations and Future Research*’

**2. Comment:** ‘On the second study, the subtitle Results line 434 The descriptive statistics of the questionnaires used are shown in Table 1 has only one table and no explanation; please see to that.’

Response: We agree that the sentence ‘The descriptive statistics of the questionnaires used are shown in Table 1’ required additional explanation. We have therefore expanded the Results section of Study 2 by adding a brief narrative summary of Table 1. This provides context for the subsequent correlational and regression analyses.

On page 20:

*‘Descriptive statistics for Study 2 are shown in Table 1. Overall, mothers reported high levels of mental load (M = 11.63, SD = 1.81; possible range 2-14). Mean conscientiousness was 3.93 (SD = 0.63) and mean neuroticism was 3.30 (SD = 0.85; both on a 1-5 scale).’*

**3. Comment:** ‘I have asked you previously to clarify your Hypotheses. Please lay it down as H1: text; H2: text and so on

for the moment, you present text as " Against this background, it is hypothesized that medium-strength positive 199

correlations will be found between mental load (perceived burden of ‘invisible’ tasks) and 200

symptoms of anxiety and depression (hypothesis 3 and 4)."

this is does not respond to the previous comments and requests since it is unclear which one is H3 and which one is H4.’

Response: Thank you for highlighting this again, and we apologize that our previous wording was not sufficiently clear. We agree that referring to “hypothesis 3 and 4” within a sentence can create ambiguity. In the revised manuscript, we have therefore reformatted the hypotheses as a numbered list (H1–Hn) and stated each hypothesis explicitly (e.g., “H3: …”, “H4: …”). In addition, we now clearly distinguish H3 (mental load and anxiety symptoms) from H4 (mental load and depressive symptoms), and we ensured that the same labels are used consistently throughout the Introduction, Results, and Discussion.

On page 10:

*‘Hypotheses*

*In the following, we summarize the study hypotheses (H1-H7).*

*H1: Invisible tasks (mental labor) are perceived as more burdensome and less equally distributed than visible tasks, particularly among mothers.*

*H2: The most frequently endorsed reasons for mental load reflect key stress characteristics of mental labor such as ongoing/never finished, boundaryless/constant thinking, low appreciation, responsibility, and unequal distribution.*

*H3: Mental load is positively associated with symptoms of generalized anxiety.*

*H4: Mental load is positively associated with depressive symptoms.*

*H5: Mental load is positively associated with neuroticism.*

*H6: Mental load is negatively associated with conscientiousness.*

*H7: Single parent status moderates the associations between neuroticism/conscientiousness and mental load.’*

**4. Comment:** ‘Please pay attention to the Journal requirements in regard to the citation style.’

Response: Thank you for this reminder. We apologize for the inconsistencies introduced during restructuring. We have now carefully checked the manuscript for full compliance with PLOS ONE’s reference requirements (numbered citation-sequence/Vancouver style). Specifically, we (i) corrected formatting issues in the text (including a bracket typo in one citation), (ii) ensured that all in-text citations appear as reference numbers in square brackets only (with instrument names presented outside the brackets), and (iii) revised the reference list to conform consistently to ICMJE/Vancouver formatting (author listing, journal format, and bibliographic details/DOIs where available).

**5. Comment:** ‘You go from Personality Traits and Mental Load to Practical implications . Maybe it would be better to explain your Results gathered for both studies, in the light of previous literature, explain if you con/in firmed the hypotheses and comment about it and further present your Conclusion future remarks and limitation.’

Response: Thank you for this helpful structural suggestion. We agree that the transition from “Personality Traits and Mental Load” to “Practical implications” was too abrupt. In the revised manuscript, we restructured the Discussion to first provide an integrated summary of the main findings from both studies, explicitly stating which hypotheses were supported, not supported, or partially supported. We then discuss the results in relation to prior literature before moving to practical implications. Finally, we added separate concluding remarks and a dedicated “Limitations and future research” section.

On page 22 (beginning of the discussion): …..*Across two studies, we found that mothers experienced invisible domestic/care tasks as particularly burdensome and unequally distributed. Regarding hypothesis testing, H1 and H2 were supported (i.e., invisible tasks were rated as more burdensome than visible tasks, and mothers reported greater perceived inequality/imbalance, with effects especially pronounced among mothers). H3–H5 were supported (i.e., higher mental load was associated with higher symptoms of anxiety and depression and with higher neuroticism). H6 was not supported (conscientiousness was not negatively related to mental load but showed a small positive association). Finally, H7 was partially supported (personality moderated the mental load–symptom association for neuroticism but not for conscientiousness). In the following, we examine these findings in more detail and discuss them in light of prior literature, implications, and limitations.*

The Discussion is now organized as follows:

- **Begin of the discussion: Integrated summary and hypothesis confirmation paragraph** (brief overview of the main findings across both studies and explicit statement of which hypotheses were supported, not supported, or partially supported)
- Burden of invisible vs. visible tasks and **reasons for the burden** (discussed in light of prior literature)
- Associations between **mental load and psychological symptoms** (discussed in light of prior literature)
- **Personality traits and moderation** (interpreted relative to existing evidence)
- **Practical implications**
- Integrated summary and conclusion
- **Limitations and future research**

These adjustments primarily involved **relabeling section headings and reordering the existing content** to ensure that results are first interpreted and contextualized before implications are presented.

**6. Comment:** ‘Please consider submitting your raw data as attached to the following version of the manuscript. Do you have the right to have and analyse it?’

Response: Thank you very much for this hint. We have uploaded the raw data on OSF: 10.17605/OSF.IO/NU5ZT

On page 3 in the manuscript:

*‘Data availability*

*The anonymized datasets are available: 10.17605/OSF.IO/NU5ZT’*

Thank you very much for reviewing our manuscript. We would like to apologize once again for the inconvenience and repetitions. We hope that we have now implemented the recommendations satisfactorily.

Best regards,

The authors
