# Supplementary material for: Mental load in women and mothers: Causes of distress, personality traits, and psychopathology
Source: PLoS One. 2026 Sep 2;21(9):e0356255. doi: 10.1371/journal.pone.0356255 (PMC13537576; doi:10.1371/journal.pone.0356255)
Supplement: Supplementary file 3 [file pone.0356255.s004.docx]

Dear Dr. Marianna Mazza, dear reviewers,

We would like to sincerely thank you, as Academic Editor, and both reviewers for your continued time, effort, and thoughtful evaluation of our manuscript entitled "Mental load in women and mothers: Causes of distress, personality traits, and psychopathology" (Manuscript ID: PONE-D-23-25403R4).

We are very grateful for the constructive and insightful comments provided throughout the review process. The reviewers' suggestions have been invaluable in further improving the scientific quality, transparency, and clarity of our manuscript. We carefully considered every comment and revised the manuscript accordingly.

In this revised version, we have addressed all remaining comments raised by the reviewers. In addition to the requested methodological, statistical, and editorial revisions, we further improved the reporting of methodological procedures, ethical aspects, and data transparency. In line with the reviewers' comments and the journal's commitment to Open Science, we also revised the manuscript to provide a clearer description of data availability and participants' rights regarding the collection and use of the data. The anonymized datasets had already been publicly registered and made available via the Open Science Framework (OSF) prior to this revision, and we now describe these procedures more explicitly within the manuscript to improve transparency and reproducibility.

Below, we provide a detailed point-by-point response to every reviewer comment. For each comment, we explain how it has been addressed in the revised manuscript and indicate the corresponding locations of the changes. All modifications are highlighted in the tracked-changes version of the manuscript.

We sincerely thank you once again for your careful consideration and for the opportunity to further improve our work. We hope that the revised manuscript is now suitable for publication in PLOS ONE.

**Reviewer 6 [R6]:**

Reviewer #6: The authors present an interesting and well-written manuscript that combines a literature review with two online survey studies examining mental load in women and mothers. Across the two cross-sectional studies, the authors compare the perceived burden associated with invisible versus visible tasks, identify the most commonly endorsed reasons underlying this burden, and investigate associations with psychological symptoms and personality traits. The findings are compelling and demonstrate, among other things, that women with children experience invisible tasks as more stressful than women without children, and that mothers perceive invisible everyday tasks as both more burdensome and less equally distributed than visible tasks. The manuscript has several methodological strengths, particularly the large sample sizes across both studies. I also believe that the practical implications considerably strengthen the contribution of the work. Overall, I enjoyed reading the manuscript and hope that the comments below may help further strengthen it.

**Authors:** *We sincerely thank the reviewer for the very positive and thoughtful evaluation of our manuscript. We greatly appreciate the encouraging assessment of the study, particularly the recognition of its methodological strengths, the large sample sizes, and the practical implications of the findings. We are grateful for the constructive suggestions, all of which have helped us to further improve the clarity, transparency, and quality of the manuscript. Below, we respond to each comment in detail.*

**1. [R6]: Abstract:** The abstract could benefit from a brief sentence describing the study design (e.g., cross-sectional online survey) and the investigated population, including a short overview of the recruitment strategy, following the sentence “Two studies aimed to systematically examine…”. It may also be helpful to include the mean age and standard deviation in parentheses alongside the sample size for each study. In addition, I would recommend briefly noting in the abstract that the manuscript also includes a literature review on mental load and related concepts.

**Authors:** *We sincerely thank the reviewer for this valuable suggestion. We agree that providing additional methodological information in the abstract improves clarity for readers. Accordingly, we have revised the abstract to explicitly state that both studies were conducted as cross-sectional online surveys, briefly describe the study populations and recruitment procedures, report the mean age and standard deviation for both samples, and indicate that the manuscript combines a literature review with two empirical studies. We believe that these revisions provide a more comprehensive overview of the study at first glance.* In addition, we made minor editorial revisions at the begin of the abstract to improve clarity, conciseness, and readability.

On page 3: e.g., *“…Following a literature review on mental load, two cross-sectional online survey studies systematically examined the burden of invisible (mental load) versus visible household and caregiving work, the causes of this burden, and associations with personality traits and psychological symptoms (affect, anxiety, depressiveness). Participants were recruited online via university mailing lists, social media, and parent forums. In Study 1 (N = 368 women; M = 35.98 years, SD = 8.87)…”*

*“…The results of Study 2 (N = 1891 mothers; M = 38.18 years, SD = 5.18)…”*

*Minor editorial revision at the beginning: “Mental load has received increasing public attention, particularly following societal changes such as the COVID-19 pandemic and shifts toward greater gender equality. However, despite its societal relevance, it remains comparatively under-researched. …”*

**2. [R6]:** Methods- Study 1: In the sentence “Paired t-tests were calculated to examine differences in the satisfaction of distribution and load of ‘invisible’ versus ‘visible’ work and a mixed ANOVA to additionally examine the effect of group (mothers with vs. without children)” (lines 308–311), I believe “mothers” should be replaced with “women” in the parentheses.

**Authors:** *We thank the reviewer for identifying this inconsistency. The wording has been corrected as suggested, and the terminology is now used consistently throughout the manuscript.*

*On page 14: “…Paired t-tests were calculated to examine differences in the satisfaction of distribution and load of ‘invisible’ versus ‘visible’ work and a mixed ANOVA to additionally examine the effect of children (women with vs. without children)….”*

**3. [R6]:** Results – Study 1: I wonder how were participant-generated reasons for mental load handled when they were entered in the open-response “other” category rather than selected from the predefined response options? I recommend adding a brief description.

**Authors:** *We thank the reviewer for this helpful suggestion. To improve methodological transparency, we have added a brief description to the Methods section explaining how responses entered in the open-text "Other" category were handled. Specifically, similar responses were reviewed and summarized into overarching categories prior to the descriptive analyses. We also added a corresponding note to Table 2 to clarify that the examples shown in parentheses represent these summarized responses.*

*On page 13: “…Regarding the burden, it was asked 5) how burdensome the ‘invisible’ and ‘visible’ tasks are perceived (0-100, 100 very burdensome) and 6) what exactly they perceived as stressful about the 6a) ‘invisible’ and 6b) ‘visible’ tasks using predefined multiple-choice response options (such as find nothing stressful about it, constant thoughts about it for ‘invisible’ tasks, amount/frequency, intensity/complexity/physical demands for ‘visible’ tasks, uneven distribution, low/lack of appreciation, lack of pay, etc.) and an additional open-text ‘Other’ category. Responses entered in the ’Other’ category were reviewed and summarized into overarching categories where appropriate prior to analysis.”*

On page 18; Notes below Table 2: *” Responses entered in the "Other" category were summarized into overarching categories where appropriate.”*

4. **[R6]:** Both studies: How long did participants take on average to complete the online surveys in Study 1 and Study 2? Were participants reimbursed for their time in any way? Additionally, was it possible for individuals to participate in both studies? I think the manuscript would benefit from these additional details.
**Authors:** We appreciate the reviewer's suggestion and have expanded the methodological description accordingly. Specifically, we now report the approximate survey duration, participant incentives, and provide additional information on the recruitment procedures. Both online surveys took approximately 15 minutes to complete. In Study 1, participants could voluntarily enter a raffle to win one of three €15 bookstore vouchers, and psychology students could receive 0.5 course credits for participation. In Study 2, no raffle or other incentive was offered (psychology students could also receive 0.5 course credits where applicable). We further clarify that, although participation in both studies cannot be completely ruled out, the studies were conducted independently and differed in their recruitment emphasis. While both studies used online recruitment, Study 2 placed a stronger emphasis on recruitment via social media platforms, making substantial overlap between the samples unlikely.

On page 11: *“Completion of the online survey took approximately 15 minutes. Participants could voluntarily enter a raffle to win one of three €15 bookstore vouchers (or psychology students could receive 0.5 course credits).”*

On pages 19/20: *“Potential participants for the Online survey ‘Mental load in mothers’ were recruited (conducted between December 13, 2022, and January 3, 2023) via university email distribution lists and, in particular, diverse social media platforms (e.g., Instagram, X) to enhance sample heterogeneity. “* …. *“Completion of the online survey took approximately 15 minutes. No raffle or other incentive was offered; however, psychology students could receive 0.5 course credits for participation where applicable.”*

Limitations on page 29: *“Although participation in both studies cannot be completely ruled out, the studies were conducted independently and used different recruitment emphases, particularly with a stronger focus on social media in Study 2. Therefore, substantial overlap between the samples is considered unlikely.”*

5. **[R6]:** Study 2: Given that only 7.6% of participants were single mothers, it may be worth briefly discussing whether the subgroup size provided sufficient power and stable estimates for moderation analyses involving single-parent status.

**Authors:** We agree that this is an important consideration. Accordingly, we have added a brief statement to the Limitations section acknowledging that the relatively small proportion of single mothers may have reduced statistical power and the stability of estimates for moderation analyses involving single-parent status.

On page 30: “*Furthermore, only a relatively small proportion of participants in Study 2 were single mothers (7.6%). Consequently, moderation analyses involving single-parent status should be interpreted with caution, as the limited subgroup size may have reduced statistical power and the stability of the corresponding estimates. Overall, it would be helpful for the generalization of the results if the samples had been more heterogeneous and representative in terms of sociodemographic variables (gender/affiliation, age/children's age, education).”*

6. **[R6]:** Discussion: I recommend switching the order of the final two sections so that “Limitations and Future Research” precedes “Integrated Summary and Conclusion.”

**Authors:** We agree that placing the Limitations and Future Research section before the Integrated Summary and Conclusion improves the overall structure and readability of the Discussion. The manuscript has been revised accordingly.

7. **[R6]:** Discussion: I also recommend noting as a limitation that the present studies did not control for the number of children or whether children had special needs, as both factors may substantially influence maternal mental load.

**Authors:** Thank you for this valuable suggestion. We agree that additional family characteristics, such as the number of children and whether a child has special needs, may influence maternal mental load. We have therefore added this point to the Limitations section and recommend that future research consider these variables to better understand factors associated with mental load.

On page 30: “*Future studies should also consider additional family characteristics, such as the number of children and whether a child has special needs, as these factors may further contribute to differences in maternal mental load.”*

Reviewer #7: General Comments
The authors made a considerable effort to address the concerns raised in the previous review round, and most points were adequately addressed, with the exception of question 6, which was only partially answered, and question 2, which requires minor correction. Furthermore, before the manuscript can be recommended for publication, it is necessary to clarify some points regarding the statistical approach adopted in both studies, as the current description raises doubts about transparency and methodological consistency.
**Authors:** We thank the reviewer for the careful evaluation of our revised manuscript and for acknowledging the substantial revisions made in response to the previous review round. We appreciate the remaining comments and have carefully addressed each point below. In particular, we have clarified the statistical procedures, revised the relevant sections of the manuscript to improve methodological transparency, and corrected the issues identified in Questions 2 and 6.

Specific Comments
1. **[R7]:** Data availability: "Do you have the right to have and analyse it?" (Previous reviewer comment #6). The authors provided the dataset on OSF, which is appreciated. However, the reviewer's question — "Do you have the right to have and analyse it?" — was not fully addressed. The response confirms where the data are available, but does not clarify the right to use the data. This information should be explicitly stated in the manuscript. A short sentence can make this information clear (e.g., ethical approval, consent, data storage, GDPR).

**Authors:** Thank you for pointing out this omission. We agree that the manuscript should explicitly clarify our authorization to collect, analyze, and share the data. We have therefore revised the Methods section to state that the studies were approved by the responsible ethics committee, that all participants provided written informed consent prior to participation, including consent for the scientific use of their anonymized data, and that data handling complied with applicable data protection regulations.

On pages 11/20: *“All participants provided written informed consent prior to participation, including consent for the scientific use of their anonymized data. Data were processed in accordance with applicable data protection regulations.”*

2. **[R7]:** Descriptive analyses report – study 2 (Previous reviewer comment #2)
Although the values from the descriptive analyses of the study were reported in Table (as well as in the text you included), it would have been appropriate to create a separate table for these values "within" study 2; in other words, one table for study 1 and another for study 2, each with its respective variables. This would facilitate the understanding and flow of information. In addition to avoiding repetition of information.

**Authors:** Thank you for this suggestion. To improve readability and facilitate the interpretation of the results, we have separated the descriptive statistics into two tables, one for Study 1 and one for Study 2.

**3., 4. And 5 [R7]:** 3. Statistical analyses — General observation (Studies 1 and 2)

Across both studies, the authors state that variables were not normally distributed, yet no specific information is provided regarding which normality test was employed (e.g., Shapiro-Wilk or Kolmogorov-Smirnov), or the outcomes of those tests. This level of detail is necessary for the reader to evaluate the appropriateness of the analytical decisions made in each study.

4. Statistical analyses — Study 1

The methods section reports the use of Paired t-tests, mixed ANOVA, and Spearman Correlations. However, given the authors' statement that the variables were not normally distributed, it is unclear whether the normality assumption was met for the parametric tests employed. This inconsistency is not addressed in either the methods or results sections.

It is acknowledged that with a large sample size (e.g., N = 354), parametric tests may be robust to normality violations in accordance with the Central Limit Theorem. However, this rationale is not explicitly stated. A clear justification for the concurrent use of parametric and non-parametric tests is necessary. Additionally, the authors do not report whether the assumptions specific to the mixed ANOVA were examined, namely sphericity and homogeneity of variances, nor whether any corrections were applied where necessary. Finally, the results section does not contextualise the analytical decisions made. Reporting statistical outcomes alone, without reference to the verified assumptions and the rationale for the chosen tests, limits the reproducibility and transparency of the findings.

5. **[R7]:** Statistical analyses — Study 2

The description of the analytical strategy in Study 2 is clearer than in Study 1, and the use of the PROCESS macro (Model 1) with bootstrap confidence intervals is appropriate and adequately justified. However, two points require clarification:

The number of bootstrap samples used in the moderation analyses is not reported. The recommended standard in the literature is 5,000 bootstrap samples, and this should be explicitly stated for reproducibility. As noted in the general observation above, the statement that variables were not normally distributed lacks specificity. Given that Spearman Correlations were chosen on this basis, identifying which variables violated normality and by which criterion they did so would strengthen the study's methodological transparency.

**Authors:** We thank the reviewer for these valuable comments regarding the statistical analyses. We agree that the description of the analytical approach required further clarification. Accordingly, we revised and expanded the Statistical Analyses sections of both studies to improve methodological transparency. In addition to addressing the specific comments raised, we also made several minor editorial revisions to improve the clarity and readability of these sections. For both studies, we have revised the Statistical Analyses sections to specify how normality was assessed (using the Shapiro–Wilk test, which indicated significant deviations from normality for several variables). We further clarified the rationale for the concurrent use of parametric and non-parametric statistical procedures. Although several variables significantly deviated from normality, the use of parametric analyses was considered appropriate because these procedures are generally regarded as robust to moderate violations of the normality assumption, particularly in larger samples, consistent with the Central Limit Theorem. Spearman rank correlations were used for the correlational analyses because several variables exhibited non-normal distributions. With respect to Study 1, we additionally clarified the assumptions underlying the mixed ANOVA. Homogeneity of variances was assessed prior to the analyses. As the within-subject factor comprised only two levels (visible vs. invisible work), the assumption of sphericity was inherently satisfied and therefore did not require formal testing or correction. To improve transparency and reproducibility, we have expanded the description of the statistical procedures accordingly. Finally, regarding the suggestion to contextualise these analytical decisions within the Results section, we have instead incorporated these clarifications into the Statistical Analyses sections, as we consider the rationale for selecting statistical procedures and verifying assumptions to better suited to the Methods section.

On page 15: *“For the statistical analyses, SPSS Statistics 23 [35] was used. Normality of the study variables was assessed using the Shapiro–Wilk test. As all study variables significantly deviated from a normal distribution, Spearman rank correlations were used to test the correlational hypotheses. In addition, paired t-tests were calculated to examine differences in the satisfaction with the distribution and load of ‘invisible’ versus ‘visible’ work, and a mixed ANOVA was conducted to additionally examine the effect of children (women with vs. without children), as these parametric procedures are generally considered robust to moderate violations of the normality assumption, particularly in larger samples, consistent with the Central Limit Theorem. Prior to conducting the mixed ANOVA, homogeneity of variances was assessed. As the within-subject factor comprised only two levels (visible vs. invisible work), the assumption of sphericity was inherently satisfied and therefore did not require formal testing. To minimize potential sources of bias, no missing values were permitted in the dataset, as the online survey required all items to be completed before submission. This ensured complete data for all variables without the need for imputation. Furthermore, to reduce the risk of error accumulation due to multiple testing, only the analyses necessary to address each specific hypothesis were conducted, and the number of statistical tests per hypothesis was kept to the minimum required. The statistical analysis plan, including the alpha level for significance testing (p < .05 for significance, p < .001 for high significance), was determined prior to data analysis. For the effect size Cohen’s d [36], the formula from Dunlop et al. [37] was used, which also includes the correlation of the variables.”*

On page 22: *“For all analyses, SPSS Statistics 23 [35] and the PROCESS macro for SPSS [39] (model 1) were used. Normality of the study variables was assessed using the Shapiro–Wilk test. As several variables significantly deviated from a normal distribution, Spearman rank correlations were used to examine bivariate associations. Moderation analyses were conducted using the PROCESS macro (Model 1) with 5,000 bootstrap samples to estimate bias-corrected 95% confidence intervals. To minimize potential sources of bias, no missing values were permitted in the dataset, as the online survey required all items to be completed before submission. This ensured complete data for all variables without the need for imputation. The statistical analysis plan, including the alpha level for significance testing (p < .05), was determined prior to data analysis.”*

*We sincerely thank the reviewers for their thoughtful and constructive comments, which have helped us improve the manuscript. We hope that the revisions satisfactorily address all comments and that the manuscript is now suitable for publication.*

*Best,*

*The authors*
