# Supplementary material for: Mental load in women and mothers: Causes of distress, personality traits, and psychopathology
Source: PLoS One. 2026 Sep 2;21(9):e0356255. doi: 10.1371/journal.pone.0356255 (PMC13537576; doi:10.1371/journal.pone.0356255)
Supplement: Peer review history

# Peer review: Decision Letter 0

**DOI:** 10.1371/journal.pone.0356255.r001

**Authors:** Ioana Gutu

2 Aug 2024

Dear Dr. Jungmann,

Please submit your revised manuscript by Sep 16 2024 11:59PM. If you will need more time than this to complete your revisions, please reply to this message or contact the journal office at plosone@plos.org. When you're ready to submit your revision, log on to https://www.editorialmanager.com/pone/ and select the 'Submissions Needing Revision' folder to locate your manuscript file.

- A rebuttal letter that responds to each point raised by the academic editor and reviewer(s). You should upload this letter as a separate file labeled 'Response to Reviewers'.
- A marked-up copy of your manuscript that highlights changes made to the original version. You should upload this as a separate file labeled 'Revised Manuscript with Track Changes'.
- An unmarked version of your revised paper without tracked changes. You should upload this as a separate file labeled 'Manuscript'.

If you would like to make changes to your financial disclosure, please include your updated statement in your cover letter. Guidelines for resubmitting your figure files are available below the reviewer comments at the end of this letter.

If applicable, we recommend that you deposit your laboratory protocols in protocols.io to enhance the reproducibility of your results. Protocols.io assigns your protocol its own identifier (DOI) so that it can be cited independently in the future. For instructions see: https://journals.plos.org/plosone/s/submission-guidelines#loc-laboratory-protocols. Additionally, PLOS ONE offers an option for publishing peer-reviewed Lab Protocol articles, which describe protocols hosted on protocols.io. Read more information on sharing protocols at https://plos.org/protocols?utm_medium=editorial-email&utm_source=authorletters&utm_campaign=protocols.

We look forward to receiving your revised manuscript.

Kind regards,

Ioana Gutu, Postdoctoral

Academic Editor

PLOS ONE

Journal Requirements:

When submitting your revision, we need you to address these additional requirements.

1. Please ensure that your manuscript meets PLOS ONE's style requirements, including those for file naming. The PLOS ONE style templates can be found at

https://journals.plos.org/plosone/s/file?id=wjVg/PLOSOne_formatting_sample_main_body.pdf and

https://journals.plos.org/plosone/s/file?id=ba62/PLOSOne_formatting_sample_title_authors_affiliations.pdf

2. Please include your full ethics statement in the ‘Methods’ section of your manuscript file. In your statement, please include the full name of the IRB or ethics committee who approved or waived your study, as well as whether or not you obtained informed written or verbal consent. If consent was waived for your study, please include this information in your statement as well.

3. Please include a separate caption for each figure in your manuscript.

[Note: HTML markup is below. Please do not edit.]

Reviewers' comments:

Reviewer's Responses to Questions

**Comments to the Author**

1. Is the manuscript technically sound, and do the data support the conclusions?

Reviewer #1: No

Reviewer #2: Yes

Reviewer #3: Partly

Reviewer #4: Yes

**********

2. Has the statistical analysis been performed appropriately and rigorously?

Reviewer #1: Yes

Reviewer #2: Yes

Reviewer #3: No

Reviewer #4: Yes

**********

3. Have the authors made all data underlying the findings in their manuscript fully available?

The PLOS Data policy

Reviewer #1: Yes

Reviewer #2: Yes

Reviewer #3: Yes

Reviewer #4: Yes

**********

4. Is the manuscript presented in an intelligible fashion and written in standard English?

Reviewer #1: Yes

Reviewer #2: Yes

Reviewer #3: No

Reviewer #4: Yes

**********

Reviewer #1: 1.What are the main claims of the paper and how significant are they for the discipline?

The paper focuses on mental load in mothers to better explain the mechanisms underying mental health suffering in this population through two studies : 1) the first study to analyse visible and invisible tasks that contribute to mental load and the relationship with mental health; 2) the second study to analyse the burden of invisible daily tasks in the context of household/care work.

2.Are the claims properly placed in the context of the previous literature? Have the authors treated the literature fairly?

The thematic of mental burden of mothers is properly placed in the context of the previous literature but the authors recongnize that previous data on this topic are scarce.

The aims are not enough clearly described.

Hypothesis are not enough justified and argued and that consititute a big methodological issue: why chosing only women in the study to consider mental burden and why exlude men ? Why do the authors do not consider other important factors that coould contribut eto mental burden such as psychiatric antecedents, traumatic events, conflict couples, children illness.... There is to much "a priori" hypothsesis" and perhaps that a qualitative study protocol could have helped to better investigate it.

3. Do the data and analyses fully support the claims? If not, what other evidence is required?

PLOS ONE encourages authors to publish detailed protocols and algorithms as supporting information online. Do any particular methods used in the manuscript warrant such treatment? If a protocol is already provided, for example for a randomized controlled trial, are there any important deviations from it? If so, have the authors explained adequately why the deviations occurred?If the paper is considered unsuitable for publication in its present form, does the study itself show sufficient potential that the authors should be encouraged to resubmit a revised version?

Despite this important topic, there is too much methodological issues to consider this paper for a publication.

4.If the paper is considered unsuitable for publication in its present form, does the study itself show sufficient potential that the authors should be encouraged to resubmit a revised version?

No

5.Are original data deposited in appropriate repositories and accession/version numbers provided for genes, proteins, mutants, diseases, etc.?

Yes, the details of the methodology are sufficient to allow the experiments to be reproduced?

6.Is any software created by the authors freely available?

Yes, there is a software created by the authors freely available.

7. Is the manuscript well organized and written clearly enough to be accessible to non-specialists?

No, the manuscript is not well organized and written clearly enough to be accessible to non-specialists, because for example they do not explain why neuroticism and perfectionnism could be in link with mental burden.

8.Is it your opinion that this manuscript contains an NIH-defined experiment of Dual Use concern?

No

Reviewer #2: -It may be a good idea to write the title "Introduction" when starting the introduction section.

-In the introduction section, more current literature can be shared about how women are affected by stressful life events and what consequences this effect has.

Data can be shared about the psychological symptoms experienced by women, especially during and after the pandemic.

The study https://doi.org/10.35365/ctjpp.21.2.13 can be used.

-The results section has very understandable and impressive data.

-I believe that it would be good to compare your target group with other similar studies in the discussion section.

You can benefit from the study https://doi.org/10.1007/s11469-022-00910-1.

- I think it is important to talk about the importance of working with gender-sensitive approaches in the detection and treatment of women's mental problems in the "Practical implications" section. this study "https://doi.org/10.35365/ctjpp.21.3.22" can be used.

Reviewer #3: The need for a professional editor or a review from a native speaker was evident already in the abstract. For example, “also” in the first sentence of the abstract implies there was something before this that this is adding onto…many issues with the writing that could be pointed out by an editor.

Why include this parenthetical comment “(preceding, parallel, following)”—I found this made the sentence much less clear because of this..

Line 60—should be amount rather than extent

I found the paragraph (lines 54-76) about different related concepts (cognitive labor, mental load) unnecessarily complex—why bring up unrelated concepts (e.g., “cognitive dimension”) rather than simply defining the words you would use--mental labor and mental load—and then using those words? Throughout the paper these selected terms should be used consistently (e.g., on line 95 mental labor is called cognitive work and on page 5 in general, or in the discussion sometimes the term “burden” is used in the place of load)

Line 83—physical symptoms

Line 158—the G*power analysis selected should be better explained and justified—a G*power analysis for a two-tailed correlational analysis with the parameters described returns a different sample size, and there are a number of analyses conducted (paired samples t-test, independent samples t-tests, and correlations). A mixed ANOVA would be the correct analysis for the findings on the right side of Figure 1 and 2, rather than this mix of individual tests. The G*power analysis could be conducted for this within-between interaction. For the correlation, it should be a two-tailed test as there was a negative predicted relationship in Study 2 between conscientiousness and mental load but a positive correlation was reported. This same answer of N = 88 is given for Study 2 without justification (Line 275). For Study 2, G*power analysis should be conducted for a moderation analysis (a regression with an interaction term) as that is the analysis used.

Line 272 “see title ‘mothers’, with divers not excluded”—typo? Unsure what this sentence means.

In Study 1, the major take-away that I think we are supposed to observe is that mothers are particularly dissatisfied with the distribution of invisible labor (which should be tested in one test, not through multiple separate independent and paired t-tests), and their perceived mental load from this is correlated with their well-being. It would be helpful to have a paragraph that summarizes this and provides a transition to Study 2. It might be helpful to have a visual model demonstrating how all of these different factors from the two studies are related (personality factors, family variables, mental load, well-being measures). This organizing framework (along with the predicted relationships) could have been pre-registered, and that would help with the haphazard feeling of the paper, along with a transition between Study 1 and Study 2. What do the authors think are the important findings of Study 1, and how is Study 2 related to these findings? The analysis for Study 2 focused only on mothers (which makes sense from the findings of Study 1) and their relationship status (but this is not introduced in the introduction to the study—there is only a single sentence introducing the idea that single mothers would be studied in Study 2 in the intro before Study 1). From the graph, it appears single mothers would report higher mental load, but the statistics are not reported for this. Is this significant? In the discussion it is reported as an effect (ll 403-404), but the statistics are not reported in the results section to know if this is actually a significant finding.

-- The children ranged in age up to 34 years old—it seems that the age range of children should be restricted to include mothers actively parenting their children as described in the discussion.

Discussion could be much stronger— both the organization and actual writing could be improved. Some sections seem more like a first draft (e.g., lines 415-432).

The following description of the significant finding (r = .14 , p < .001) is problematic: “Regarding conscientiousness, contrary to our hypothesis, we found a positive relationship with mental load, but with a very small effect (probably not significant in a smaller sample).” Yes, a correlation around r = .10 is considered a small effect size, but researchers cannot assert that a significant effect is not really significant (just as one can’t say an insignificant effect would be significant with a larger sample size).

Notes would be helpful for the figures (e.g., which study the data are from, what the error bars represent, if the low/high categories are a median split or +/-1 SD etc). In Figure 3, it would be helpful to use the term mothers rather than parents to be clear that this is only women.

Reviewer #4: Thank you for writing such an interesting paper. It was a pleasure to read it. As a reader, it would be great to understand your sample's socio-cultural contexts to get a better perspective on their mental load. Since mothers are operating within the societal contexts and each context has its own boundaries, and implications on a mother's mental load, requesting authors to include this bit in the methodology as well as weave it in the discussion.

**********

what does this mean?). If published, this will include your full peer review and any attached files.

If you choose “no”, your identity will remain anonymous but your review may still be made public.

**Do you want your identity to be public for this peer review?**  For information about this choice, including consent withdrawal, please see our Privacy Policy

Reviewer #1: No

Reviewer #2: No

Reviewer #3: No

Reviewer #4: **Yes:s** Ketoki Mazumdar

**********

[NOTE: If reviewer comments were submitted as an attachment file, they will be attached to this email and accessible via the submission site. Please log into your account, locate the manuscript record, and check for the action link "View Attachments". If this link does not appear, there are no attachment files.]

While revising your submission, please upload your figure files to the Preflight Analysis and Conversion Engine (PACE) digital diagnostic tool, https://pacev2.apexcovantage.com/. PACE helps ensure that figures meet PLOS requirements. To use PACE, you must first register as a user. Registration is free. Then, login and navigate to the UPLOAD tab, where you will find detailed instructions on how to use the tool. If you encounter any issues or have any questions when using PACE, please email PLOS at figures@plos.org. Please note that Supporting Information files do not need this step.

# Author comment: Author response to Decision Letter 1

**DOI:** 10.1371/journal.pone.0356255.r002

23 Sep 2024

Dear Dr. Ioana Gutu,

dear reviewers,

Thank you very much for your letter, and for the very helpful reviews of our manuscript titled ‘Mental load in women and mothers: Causes of distress, personality traits, and psychopathology’ (PONE-D-23-25403).

We thank you and the reviewer for the thoughtful and constructive feedback and suggestions for improving the manuscript. We have carefully revised the manuscript as recommended (one version with tracked changes) and address the comments point by point below.

Comments editor:

1.) Please ensure that your manuscript meets PLOS ONE's style requirements, including those for file naming. The PLOS ONE style templates can be found at

https://journals.plos.org/plosone/s/file?id=wjVg/PLOSOne_formatting_sample_main_body.pdf and

https://journals.plos.org/plosone/s/file?id=ba62/PLOSOne_formatting_sample_title_authors_affiliations.pdf

[AU]: In the revised version, we have checked and revised the style requirements, such as levels and formatting of headings and figure citations/file naming, and we have deleted information of the corresponding author (only E-Mail).

2) Please include your full ethics statement in the ‘Methods’ section of your manuscript file. In your statement, please include the full name of the IRB or ethics committee who approved or waived your study, as well as whether or not you obtained informed written or verbal consent. If consent was waived for your study, please include this information in your statement as well.

[AU]: Thank you for the helpful comment. We have inserted additional information in the Method section.

‘The ethics committee of the Psychological Institute of the Johannes Gutenberg-University Mainz has approved the study project.’ (on pages 8 and 15)

On pages 7 (Study 1) and 14 (Study 2) in the first submission (and now the revision), we described the written informed consent (‘After providing written informed consent…, ‘Written informed consent was required prior to participation.’).

3) Please include a separate caption for each figure in your manuscript.

[AU]: Thank you for this hint. As recommended, we have inserted captions for each figure (see pages 11, 12 and 16).

Comments reviewer

Reviewer #1:

1. What are the main claims of the paper and how significant are they for the discipline? The paper focuses on mental load in mothers to better explain the mechanisms underying mental health suffering in this population through two studies : 1) the first study to analyse visible and invisible tasks that contribute to mental load and the relationship with mental health; 2) the second study to analyse the burden of invisible daily tasks in the context of household/care work.

2.Are the claims properly placed in the context of the previous literature? Have the authors treated the literature fairly?

The thematic of mental burden of mothers is properly placed in the context of the previous literature but the authors recongnize that previous data on this topic are scarce.

The aims are not enough clearly described.

Hypothesis are not enough justified and argued and that consititute a big methodological issue: why chosing only women in the study to consider mental burden and why exlude men ? Why do the authors do not consider other important factors that coould contribut eto mental burden such as psychiatric antecedents, traumatic events, conflict couples, children illness.... There is to much "a priori" hypothsesis" and perhaps that a qualitative study protocol could have helped to better investigate it.

[AU]: Thank you for the positive feedback mentioned in the introduction. As recommended, we have specified the objectives in the revised version and derived hypotheses that are theoretically better founded. We integrated theories and derived our hypotheses from them. In addition, we conducted a new literature search (e.g., meta-analysis by Ren et al., 2024). We can fully understand your questions, would like to explain them here and have expanded the discussion and limitations in this regard. The study objectives were a compromise between building on the previous ones (especially the target group) and expanding the previous findings (e.g,. to specify mental load and mental health in general for women here or to derive burnout research from the area of mental load). We fully agree that this should be described in the manuscript for a better understanding.

“These characteristics are supported by the Transactional Stress Model [13], which states that the subjective evaluation of a situation/event as stressful is crucial, as are the individual resources available to cope with the situation.” (On page 5)

“A recent systematic review [18] identified 26 articles on parental burnout and categorized associated factors according to the Ecological Systems Theory (EST) [19]. In particular, factors at the individual microsystem level, e.g., sociodemographic data, personality factors, and anxiety/depression, were found to be associated with parental burnout. The latter (mental health problems) e.g., due to the overlap of dysfunction of the hypothalamic-pituitary-adrenal axis (stress axis). The (risk) factors associated with parental burnout may resemble the mental workload, although there is a lack of systematic scientific research into the reasons why mental labor is perceived as stressful and how mental load is related to mental health.” (on pages 5 and 6)

“In this paper, we aim to fill these research gaps with two studies (risk factors of mental load, associations of mental load with personality traits and mental health symptoms).” (on page 6)

“Given the characteristics of mental load and the and the overlap with parental burnout, we assumed that the ‘invisible’ tasks are perceived as more burdensome than the ‘visible’ ones and that the perceived burden of ‘invisible’ tasks is positively related to anxiety and depressive symptoms.” (on pages 5 and 7)

“In the context of parental burnout, there is also the assumption that neuroticism is associated with deficits in emotion regulation and that emotionally unstable parents react more strongly to life events, which can increase the likelihood of parental burnout [18]. So similar to findings in burnout and stress research [18, 21, 25, 26], it could also be hypothesized that neuroticism is positively related to mental load.” (on page 7)

“Our study has a specific focus on mental load, personality traits and psychological symptoms in women and mothers. In terms of gender, the studies are one-sided (social role as a woman) and cannot be generalized to men (social role/affiliated)/non-binary/diverse. In addition, many other potential risk factors were not examined, such as previous psychiatric illnesses, relationship quality/couple conflicts, physical illnesses of family members, which should be considered in further studies.” (on page 22)

3. Do the data and analyses fully support the claims? If not, what other evidence is required?

PLOS ONE encourages authors to publish detailed protocols and algorithms as supporting information online. Do any particular methods used in the manuscript warrant such treatment? If a protocol is already provided, for example for a randomized controlled trial, are there any important deviations from it? If so, have the authors explained adequately why the deviations occurred?If the paper is considered unsuitable for publication in its present form, does the study itself show sufficient potential that the authors should be encouraged to resubmit a revised version?

Despite this important topic, there is too much methodological issues to consider this

paper for a publication.

[AU]: Thank you for your feedback. The aim of our studies was to advance the new research field of mental load and, based on our results, to encourage further research in this area. In our limitations, we discuss weaknesses of the studies, and as recommended, we have expanded them.

4.If the paper is considered unsuitable for publication in its present form, does the study itself show sufficient potential that the authors should be encouraged to resubmit a revised version?

No; 5. Are original data deposited in appropriate repositories and accession/version numbers provided for genes, proteins, mutants, diseases, etc.?

Yes, the details of the methodology are sufficient to allow the experiments to be reproduced? ; 6.Is any software created by the authors freely available?

Yes, there is a software created by the authors freely available.

7. Is the manuscript well organized and written clearly enough to be accessible to non-specialists?

No, the manuscript is not well organized and written clearly enough to be accessible to non-specialists, because for example they do not explain why neuroticism and perfectionnism could be in link with mental burden.

[AU]: We would like to thank the reviewer for suggesting that we explain the hypotheses regarding the connection between personality traits (neuroticism and conscientiousness) and mental load in more detail. We have made additions to the revised version (see also point 1).

“Study 2 addressed the relationship between the personality traits neuroticism as well as conscientiousness and mental labor in the context of family. Individuals with higher neuroticism are considered to be more emotionally unstable, sensitive, and tend to become more easily unbalanced under stress. In the context of parental burnout, there is also the assumption that neuroticism is associated with deficits in emotion regulation and that emotionally unstable parents react more strongly to life events, which can increase the likelihood of parental burnout [18]., Sso similar to findings in burnout and stress research [18, 21, 25, 26], it could also be hypothesized that neuroticism is positively related to mental load. Regarding conscientiousness, individuals with higher levels are thought to be more organized, disciplined, and have better time management [21], and negative associations have been found between conscientiousness and parental and job burnout [21, 25]. Accordingly, we assume a negative correlation between conscientiousness and mental load in the context of family.” (on page 7)

8.Is it your opinion that this manuscript contains an NIH-defined experiment of Dual Use concern?

No

Reviewer #2:

-It may be a good idea to write the title "Introduction" when starting the introduction section.

[Au]: Thank you very much, we have added this heading.

-In the introduction section, more current literature can be shared about how women are affected by stressful life events and what consequences this effect has.

Data can be shared about the psychological symptoms experienced by women, especially during and after the pandemic.

The study https://doi.org/10.35365/ctjpp.21.2.13 can be used.

[Au]: We thank you very much for the recommendation of this very interesting article, which we were pleased to expand. In addition, we had carried out a literature search and added current literature (e.g. Ren et al., 2004, systematic review on parental burnout).

“During the COVID-19 pandemic (particularly during the lockdown), much of the double burden (housework, care work including homeschooling, and paid work, respectively) fell on women and mothers, who reported increased levels of burnout, loneliness, anxiety and depression during this time [12, 13].” (on page 4)

-The results section has very understandable and impressive data.

[Au]: Thank you for the positive feedback. We are very happy to hear that.

-I believe that it would be good to compare your target group with other similar studies in the discussion section.

You can benefit from the study https://doi.org/10.1007/s11469-022-00910-1.

[Au]: Thank you. We absolutely agree that this is a very interesting addition to our discussion:

“Our findings are consistent with the study by Kaplan [41], who found that, in housewives, co-dependence (the tendency to prioritize the needs of others over one's own) was associated with psychological symptoms such as anxiety and depression. Furthermore, it clarifies or expands on another possible toxic feature of mental load, which was mentioned above as including responsibility, emotional labor, and relationship work.” (on page 20)

- I think it is important to talk about the importance of working with gender-sensitive approaches in the detection and treatment of women's mental problems in the "Practical implications" section. this study "https://doi.org/10.35365/ctjpp.21.3.22" can be used.

[AU]: We agree with you that this is a very important topic, which we have added to. In addition, based on the recommendation of another reviewer, we have also expanded the limitation.

“Since (social) gender plays a crucial role in determining the living environment and life tasks (such as the take-over of care work) in many cultures, it is important to keep these aspects in mind when considering interventions or therapies. For example, Kaplan [45] suggests a feminist therapy that specifically addresses the needs of women (e.g,, feelings of guilt).” (on page 22)

“In terms of gender, the studies are one-sided (social role as a woman) and cannot be generalized to men (social role/affiliated)/non-binary/diverse.” (on page 22)

Reviewer #3: The need for a professional editor or a review from a native speaker was evident already in the abstract. For example, “also” in the first sentence of the abstract implies there was something before this that this is adding onto…many issues with the writing that could be pointed out by an editor.

[AU]: Thank you for your extremely helpful feedback. We have reviewed the wording. We have deleted “also” in the abstract.

Why include this parenthetical comment “(preceding, parallel, following)”—I found this made the sentence much less clear because of this..

[Au]: We absolutely agree with you that the sentence is easier to understand without the brackets, and we have deleted them.

Line 60—should be amount rather than extent

[Au]: Thank you very much, we have exchanged words.

I found the paragraph (lines 54-76) about different related concepts (cognitive labor, mental load) unnecessarily complex—why bring up unrelated concepts (e.g., “cognitive dimension”) rather than simply defining the words you would use--mental labor and mental load—and then using those words? Throughout the paper these selected terms should be used consistently (e.g., on line 95 mental labor is called cognitive work and on page 5 in general, or in the discussion sometimes the term “burden” is used in the place of load)

[Au]: We thank the reviewer for the helpful recommendations. We have now simplified the introduction with the definitions and standardized the use of terms.

Deleted: “According to Sweller et al. [6], cognitive load refers to the load on working memory, whereby the capacity for the amount of information is limited. Mental workload, as defined by Young and Stanon [7, p. 507], describes the extent of "attentional resources required to meet both objective and subjective performance criteria". (on page 3)

“Dean et al. [3] describe three characteristics, in particular, that contribute to the stressful and exhausting nature of mental laborcognitive work: 1. these tasks and duties are ‘invisible’ (e.g., little valued), 2. they are boundaryless (e.g., flowing or interfering with falling asleep, paid work, or leisure), 3. they are ongoing (e.g., few short- or longer-term breaks).” (on page 5)

Discussion, for example:

“Mothers in particular perceived an uneven distribution and mental load, with about one-third citing perfectionism as the cause of mental load and about half citing not being able to break out of social roles.” (on page 21)

Line 83—physical symptoms

[Au]: Thank you for pointing this out; we have reworded it.

Line 158—the G*power analysis selected should be better explained and justified—a G*power analysis for a two-tailed correlational analysis with the parameters described returns a different sample size, and there are a number of analyses conducted (paired samples t-test, independent samples t-tests, and correlations). A mixed ANOVA would be the correct analysis for the findings on the right side of Figure 1 and 2, rathe

Attachment Submitted filename: Response to Reviewers.docx

# Peer review: Decision Letter 1

**DOI:** 10.1371/journal.pone.0356255.r003

**Authors:** Ioana Gutu

15 Nov 2024

Dear Dr. Jungmann,

Please submit your revised manuscript by Dec 30 2024 11:59PM. If you will need more time than this to complete your revisions, please reply to this message or contact the journal office at plosone@plos.org. When you're ready to submit your revision, log on to https://www.editorialmanager.com/pone/ and select the 'Submissions Needing Revision' folder to locate your manuscript file.

- A rebuttal letter that responds to each point raised by the academic editor and reviewer(s). You should upload this letter as a separate file labeled 'Response to Reviewers'.
- A marked-up copy of your manuscript that highlights changes made to the original version. You should upload this as a separate file labeled 'Revised Manuscript with Track Changes'.
- An unmarked version of your revised paper without tracked changes. You should upload this as a separate file labeled 'Manuscript'.

If applicable, we recommend that you deposit your laboratory protocols in protocols.io to enhance the reproducibility of your results. Protocols.io assigns your protocol its own identifier (DOI) so that it can be cited independently in the future. For instructions see: https://journals.plos.org/plosone/s/submission-guidelines#loc-laboratory-protocols. Additionally, PLOS ONE offers an option for publishing peer-reviewed Lab Protocol articles, which describe protocols hosted on protocols.io. Read more information on sharing protocols at https://plos.org/protocols?utm_medium=editorial-email&utm_source=authorletters&utm_campaign=protocols.

We look forward to receiving your revised manuscript.

Kind regards,

Ioana Gutu, Postdoctoral

Academic Editor

PLOS ONE

Journal Requirements:

Please review your reference list to ensure that it is complete and correct. If you have cited papers that have been retracted, please include the rationale for doing so in the manuscript text, or remove these references and replace them with relevant current references. Any changes to the reference list should be mentioned in the rebuttal letter that accompanies your revised manuscript. If you need to cite a retracted article, indicate the article’s retracted status in the References list and also include a citation and full reference for the retraction notice.

[Note: HTML markup is below. Please do not edit.]

Reviewers' comments:

Reviewer's Responses to Questions

**Comments to the Author**

Reviewer #2: All comments have been addressed

Reviewer #5: (No Response)

**********

2. Is the manuscript technically sound, and do the data support the conclusions?

Reviewer #2: Yes

Reviewer #5: Partly

**********

3. Has the statistical analysis been performed appropriately and rigorously?

Reviewer #2: Yes

Reviewer #5: Yes

**********

4. Have the authors made all data underlying the findings in their manuscript fully available?

The PLOS Data policy

Reviewer #2: Yes

Reviewer #5: Yes

**********

5. Is the manuscript presented in an intelligible fashion and written in standard English?

Reviewer #2: Yes

Reviewer #5: Yes

**********

Reviewer #2: Congrat to all the authour. All the revisions you made are great. I believe that your work will make important contributions to the literature.

Reviewer #5: Dear Author,

Your manuscript has a very interesting and carefully chosen subject that would clearly benefit practice and literature when published.

However, the current version does not currently meet the criteria for being published, reason for which the next issues need to be taken care of.

First, the abstract and the entire manuscript must use an impersonal style.

Additionally, the introduction needs to be improved as to better highlight the research problem, as well as the entire structure of the manuscript, by adding a final paragraph where you explain this structure.

As for the literature review, although you comply with the general rule of analyzing in pairs the variables, it is rather scarce. Please improve this part, which is also reflected within your references list. Please make sure that you consider and explain each variable in the light of previous literature. Also, from your literature review must result a number of hypotheses that you describe in the light of previous literature now, test further and make a special subchapter later and discuss the accepted/rejected hypotheses and the results in the light of previous literature.

The Methodology chapter needs to be checked again and mention for each scale and survey/questionnaire used that measures and their format (e.g. Likert 4 5 7 items and so on).

Also clarify the software that you have been using and justify your choice.

Moreover, cite and describe all the thresholds for your analysis.

Please describe how are you avoiding bias within your data curation, analysis method use and how do you perform the generalization of your results.

Please add a final section where you display your final remarks in regard to your research subject, findings, previous literature remarks.

Best regards,

**********

what does this mean?). If published, this will include your full peer review and any attached files.

If you choose “no”, your identity will remain anonymous but your review may still be made public.

**Do you want your identity to be public for this peer review?**  For information about this choice, including consent withdrawal, please see our Privacy Policy

Reviewer #2: No

Reviewer #5: No

**********

[NOTE: If reviewer comments were submitted as an attachment file, they will be attached to this email and accessible via the submission site. Please log into your account, locate the manuscript record, and check for the action link "View Attachments". If this link does not appear, there are no attachment files.]

While revising your submission, please upload your figure files to the Preflight Analysis and Conversion Engine (PACE) digital diagnostic tool, https://pacev2.apexcovantage.com/. PACE helps ensure that figures meet PLOS requirements. To use PACE, you must first register as a user. Registration is free. Then, login and navigate to the UPLOAD tab, where you will find detailed instructions on how to use the tool. If you encounter any issues or have any questions when using PACE, please email PLOS at figures@plos.org

# Author comment: Author response to Decision Letter 2

**DOI:** 10.1371/journal.pone.0356255.r004

7 Feb 2025

Dear Dr. Ioana Gutu,

dear reviewers,

Thank you very much for your letter and the further suggestions for improvement of our manuscript titled ‘Mental load in women and mothers: Causes of distress, personality traits, and psychopathology’ (PONE-D-23-25403).

We thank the reviewers for checking our revised version and are pleased to hear that most points (especially those of reviewer 2) have been implemented to their full satisfaction.

The comments from reviewer 5 are very helpful and we have revised the manuscript according to the recommendations. Since this mainly concerns point 6 (point 2 is seen as partially fulfilled and associated with point 6), we will provide a summary of our responses below (respectively, broken down in detail by content points within 6.) .

I. Journal Requirements:

Please review your reference list to ensure that it is complete and correct. If you have cited papers that have been retracted, please include the rationale for doing so in the manuscript text, or remove these references and replace them with relevant current references. Any changes to the reference list should be mentioned in the rebuttal letter that accompanies your revised manuscript. If you need to cite a retracted article, indicate the article’s retracted status in the References list and also include a citation and full reference for the retraction notice.

[Author]: We have marked all changes regarding the literature in the text and the list of literature and have harmonized the text and the list of literature at the end.

II. Reviewers' comments:

1. If the authors have adequately addressed your comments raised in a previous round of review and you feel that this manuscript is now acceptable for publication, you may indicate that here to bypass the “Comments to the Author” section, enter your conflict of interest statement in the “Confidential to Editor” section, and submit your "Accept" recommendation.

Reviewer #2: All comments have been addressed

Reviewer #5: (No Response)

2. Is the manuscript technically sound, and do the data support the conclusions?

The manuscript must describe a technically sound piece of scientific research with data that supports the conclusions. Experiments must have been conducted rigorously, with appropriate controls, replication, and sample sizes. The conclusions must be drawn appropriately based on the data presented.

Reviewer #2: Yes

Reviewer #5: Partly

3. Has the statistical analysis been performed appropriately and rigorously?

Reviewer #2: Yes

Reviewer #5: Yes

4. Have the authors made all data underlying the findings in their manuscript fully available?

The PLOS Data policy requires authors to make all data underlying the findings described in their manuscript fully available without restriction, with rare exception (please refer to the Data Availability Statement in the manuscript PDF file). The data should be provided as part of the manuscript or its supporting information, or deposited to a public repository. For example, in addition to summary statistics, the data points behind means, medians and variance measures should be available. If there are restrictions on publicly sharing data—e.g. participant privacy or use of data from a third party—those must be specified.

Reviewer #2: Yes

Reviewer #5: Yes

5. Is the manuscript presented in an intelligible fashion and written in standard English? PLOS ONE does not copyedit accepted manuscripts, so the language in submitted articles must be clear, correct, and unambiguous. Any typographical or grammatical errors should be corrected at revision, so please note any specific errors here.

Reviewer #2: Yes

Reviewer #5: Yes

6. Review Comments to the Author

Please use the space provided to explain your answers to the questions above. You may also include additional comments for the author, including concerns about dual publication, research ethics, or publication ethics. (Please upload your review as an attachment if it exceeds 20,000 characters)

Reviewer #2: Congrat to all the authour. All the revisions you made are great. I believe that your work will make important contributions to the literature.

Reviewer #5: Dear Author,

Your manuscript has a very interesting and carefully chosen subject that would clearly benefit practice and literature when published.

However, the current version does not currently meet the criteria for being published, reason for which the next issues need to be taken care of.

First, the abstract and the entire manuscript must use an impersonal style.

Additionally, the introduction needs to be improved as to better highlight the research problem, as well as the entire structure of the manuscript, by adding a final paragraph where you explain this structure.

As for the literature review, although you comply with the general rule of analyzing in pairs the variables, it is rather scarce. Please improve this part, which is also reflected within your references list. Please make sure that you consider and explain each variable in the light of previous literature. Also, from your literature review must result a number of hypotheses that you describe in the light of previous literature now, test further and make a special subchapter later and discuss the accepted/rejected hypotheses and the results in the light of previous literature.

The Methodology chapter needs to be checked again and mention for each scale and survey/questionnaire used that measures and their format (e.g. Likert 4 5 7 items and so on).

Also clarify the software that you have been using and justify your choice.

Moreover, cite and describe all the thresholds for your analysis.

Please describe how are you avoiding bias within your data curation, analysis method use and how do you perform the generalization of your results.

Please add a final section where you display your final remarks in regard to your research subject, findings, previous literature remarks.

[Author]: Thank you for your feedback. We are pleased to hear that most of the points are viewed positively. Regarding point 6, we would like to go into the revisions in detail here, in line with the recommendations of reviewer 5.

a) Impersonal style: Thank you for pointing this out. We have checked the abstract and the complete manuscript and used an impersonal style (e.g., “we” replaced/rewritten).

e.g., abstract: “The results of Study 2 (N = 1891 mothers) showed, that mental load is positively related to neuroticism and conscientiousness, with the association of mental load and neuroticism moderated by being a single mother.”

e.g., on pages 6/7: “So, this paper is intended to help closing these research gaps.”

e.g., on page 7: “In Study 1, the focus is additionally on examining the relationship between ML and psychological symptoms (anxiety and depressive symptoms). …”

e.g., on page 7: “…it was assumed that the ‘invisible’ tasks are perceived as more burdensome than the ‘visible’ ones and that the perceived burden of ‘invisible’ tasks is positively related to anxiety and depressive symptoms.”

e.g., on page 8: “Accordingly, a negative correlation between conscientiousness and mental load in the context of family was assumed.”

b) Introduction: As recommended, we emphasized the research problem more strongly in revision 2 and added a section in which we explain the structure of the manuscript.

On page 6: “Mental labor can be particularly stressful for women, can be associated with poorer mental health, and can restrict other areas of life [8, 9, 24]. In public, mental load has become a hot topic, but in contrast, it has hardly been scientifically investigated [24]. Therefore, it is relevant to systematically examine which aspects of mental labor exactly constitute the burden, what role personality factors play (e.g., as possible risk factors), and how ML is related to psychopathological symptoms. The aim is to identify stressors and risks (and interactions, if applicable) in order to develop a well-founded approach to reducing them (i.e., prevention and intervention approaches) in a second step.”

On page 7: “This article comprises two studies, which are reported consecutively. In both studies, the perceived burden of ‘visible’ (physical) tasks compared to ‘invisible’ tasks (cognitive/mental labor) in the context of family and the reasons for the burden was systematically examined (Study 2 for testing in a larger sample). …. In study 1, the focus is additionally on examining the relationship between ML and psychological symptoms (anxiety and depressive symptoms). … In Study 2, the focus is additionally on the relationship between the personality traits neuroticism as well as conscientiousness and mental labor in the context of family.”

c) Each variable: Thank you for the constructive feedback. In revision 2, we have addressed the individual variables more explicitly and in more detail in the introduction/theoretical background. In addition, the hypotheses/results were discussed more strongly in the context of this literature.

e.g., on page 7: “The reasons and characteristics of why mental labor is a burden are to be identified. Specifically, investigation of the causes should confirm the characteristics stated in the model of Dean et al. [3] as well as those empirically identified so far (e.g., responsibility, time commitment).”

e.g., on page 7: “Based on biological and psychological models (see above HPA-axis, Transactional Stress Model) and previous findings that mental labor is associated with exhaustion/excessive demands, negative affect and [16], in a recently published study, with depressive symptoms [24], medium-strength positive correlations between ML (perceived burden of 'invisible' tasks) and anxiety and depressive symptoms are expected.”

The hypotheses on the personality traits of neuroticism and conscientiousness should now also be more comprehensible as a result of the restructuring of the hypothesis section.

On page 8: “In Study 2, the focus is additionally onaddressed the relationship between the personality traits neuroticism as well as conscientiousness and mental labor in the context of family (traits, that are particularly relevant in the context of parental burnout, see above [20]). Individuals with higher neuroticism are considered to be more emotionally unstable, sensitive, and tend to become more easily unbalanced under stress. In the context of parental burnout, there is also the assumption that neuroticism is associated with deficits in emotion regulation and that emotionally unstable parents react more strongly to life events, which can increase the likelihood of parental burnout [17]. So similar to findings in burnout and stress research [17, 20, 25, 26], it could also be hypothesized that neuroticism is positively related to mental load. Regarding conscientiousness, individuals with higher levels are thought to be more organized, disciplined, and have better time management [20], and negative associations have been found between conscientiousness and parental and job burnout [20, 25].”

On page 21: “In an earlier study [40] , it was shown that ML is associated with feelings of being overwhelmed and overburdened, which may be related to or contribute to anxiety. The results of the present studies show this specifically for generalized anxiety. Regarding depressive symptoms, a recent study [24] also found that the distribution of cognitive domestic labor is related to depressive symptoms. Complementing this, our study showed that the perceived burden of unpaid/invisible household tasks is related to depressive symptoms.”

d) Method: The methods section was carefully checked and the characteristics of the measuring instruments were added (e.g., response format and response anchor). In addition, information on the software was added and the use of statistical methods was more strongly justified (the limitations focus more on the aspect of generalizability).

On page 10: “..(adjectives such as ‘enthusiastic’ and ‘nervous’, 5-point Likert scale ranging from 1=very little/not at all to 5=extremely).”

On page 10: “(e.g., ‘Worrying too much about different things’, 4-point Likert scale from 0=not at all to 3=nearly every day).”

On page 11: “(‘little interest or pleasure in doing thing’ and ‘feeling, down, depressed, or hopeless’, 4-point Likert scale from 0=never to 3=almost every day).”

On page 17: “Each dimension consists of 4 items which can be answered using a 5-point Likert scale (1=disagree a lot to 5=agree al lot).”

On page 11: “For the statistical analyses, the software SPSS Statistics 23 was used. During the online survey, questions could not be skipped, so there were no missing values.”

On page 17: “For all analyses, SPSS Statistics software version 23 was used. ….. In addition, two moderation analyses were calculated using the PROCESS macro for SPSS [34] to examine whether the associations between neuroticism/conscientiousness and mental load are moderated by single parent status.”

On pages 23/24: “The women in study 1 and 2 had a higher level of education compared to the general German population, so the study is not representative in this respect. …. Overall, it would be helpful for the generalization of the results if the samples had been more heterogeneous and representative in terms of sociodemographic variables (gender/affiliation, age/children's age, education).”

e) Results: As recommended, we added statistical characteristics (the range of values/thresholds). In Table 1, the range values were added. It was noticed that the ranges were from 1-101 (because the survey platform starts with 1 instead of zero by default). This was corrected so that the mean values were also corrected by 1 in each case. We have also adjusted Figures 1 and 2 (average values reduced by one), all other results remain the same. We apologize for this and thank you for pointing it out.

f) Conclusion: We fully agree with the expert that final remarks can be made clearer and more extensive and have added this in revision 2.

On page 25: “The two present studies with women and mothers were the first to systematically examine the burden of invisible care/household tasks (mental load) compared with visible ones, the causes of mental load, and associations with personality traits and psychopathology. … In comparison to previous studies, mental load was assessed based on the transactional stress model as the subjective burden of invisible household/care tasks, and previous studies were extended to include additional factors and their interaction. ... Regarding the investigation of affect and psychopathological symptoms, this study extended findings from burnout and stress research specifically for the area of mental load. ….”

We hope that we have implemented the revisions to your satisfaction.

Sincerely,

the authors

# Peer review: Decision Letter 2

**DOI:** 10.1371/journal.pone.0356255.r005

**Authors:** Ioana Gutu

15 Mar 2025

Dear Dr. Jungmann,

Thank you for submitting your manuscript to PLOS ONE. After careful consideration, we feel that it has merit but does not fully meet PLOS ONE’s publication criteria as it currently stands. Therefore, we invite you to submit a revised version of the manuscript that addresses the points raised during the review process.

Please consider all the aspects of the Review Letter in detail.

Please submit your revised manuscript by Apr 29 2025 11:59PM. If you will need more time than this to complete your revisions, please reply to this message or contact the journal office at plosone@plos.org

- A rebuttal letter that responds to each point raised by the academic editor and reviewer(s). You should upload this letter as a separate file labeled 'Response to Reviewers'.
- A marked-up copy of your manuscript that highlights changes made to the original version. You should upload this as a separate file labeled 'Revised Manuscript with Track Changes'.
- An unmarked version of your revised paper without tracked changes. You should upload this as a separate file labeled 'Manuscript'.

If applicable, we recommend that you deposit your laboratory protocols in protocols.io to enhance the reproducibility of your results. Protocols.io assigns your protocol its own identifier (DOI) so that it can be cited independently in the future. For instructions see: https://journals.plos.org/plosone/s/submission-guidelines#loc-laboratory-protocols. Additionally, PLOS ONE offers an option for publishing peer-reviewed Lab Protocol articles, which describe protocols hosted on protocols.io. Read more information on sharing protocols at https://plos.org/protocols?utm_medium=editorial-email&utm_source=authorletters&utm_campaign=protocols.

We look forward to receiving your revised manuscript.

Kind regards,

Ioana Gutu, Postdoctoral

Academic Editor

PLOS ONE

Journal Requirements:

Please review your reference list to ensure that it is complete and correct. If you have cited papers that have been retracted, please include the rationale for doing so in the manuscript text, or remove these references and replace them with relevant current references. Any changes to the reference list should be mentioned in the rebuttal letter that accompanies your revised manuscript. If you need to cite a retracted article, indicate the article’s retracted status in the References list and also include a citation and full reference for the retraction notice.

[Note: HTML markup is below. Please do not edit.]

Reviewers' comments:

Reviewer's Responses to Questions

**Comments to the Author**

Reviewer #5: (No Response)

**********

2. Is the manuscript technically sound, and do the data support the conclusions?

Reviewer #5: No

**********

3. Has the statistical analysis been performed appropriately and rigorously?

Reviewer #5: No

**********

4. Have the authors made all data underlying the findings in their manuscript fully available?

The PLOS Data policy

Reviewer #5: No

**********

5. Is the manuscript presented in an intelligible fashion and written in standard English?

Reviewer #5: Yes

**********

Reviewer #5: Dear Author,

As previously suggested, your manuscript covers an interesting study area.

However, you need to carefully consider each aspect required by the reviewers, since only in this case, the research article would be considered for publication.

as previously stated,

- the introduction needs to be improved as to better highlight the research problem, as well as the entire structure of the manuscript; maybe it is an error, but for the moment your introduction has one paragraph where you do not explain the research subject, the gap, the RQ nor a final paragraph explaining the entire manuscriptstructire;

- the literature review, although you comply with the general rule of analyzing in pairs the variables, it is rather scarce, a fact reflected within your very few references.

- Please improve this part, which is also reflected within your references list.

- Please make sure that you consider and explain each variable in the light of previous literature.

- Also, from your literature review must result a number of hypotheses that you describe in the light of previous literature now, test further and make a special subchapter later and discuss the accepted/rejected hypotheses and the results in the light of previous literature.

- The Methodology chapter needs to be checked again and mention for each scale and survey/questionnaire used that measures and their format (e.g. Likert 4 5 7 items and so on).

- clarify the software that you have been using and justify your choice.

- cite and describe all the thresholds for your analysis.

- describe how are you avoiding bias within your data curation, analysis method use and how do you perform the generalization of your results.

- add a final section where you display your final remarks in regard to your research subject, findings, previous literature remarks.

Best regard

**********

what does this mean?). If published, this will include your full peer review and any attached files.

If you choose “no”, your identity will remain anonymous but your review may still be made public.

**Do you want your identity to be public for this peer review?**  For information about this choice, including consent withdrawal, please see our Privacy Policy

Reviewer #5: No

**********

[NOTE: If reviewer comments were submitted as an attachment file, they will be attached to this email and accessible via the submission site. Please log into your account, locate the manuscript record, and check for the action link "View Attachments". If this link does not appear, there are no attachment files.]

While revising your submission, please upload your figure files to the Preflight Analysis and Conversion Engine (PACE) digital diagnostic tool, https://pacev2.apexcovantage.com/. PACE helps ensure that figures meet PLOS requirements. To use PACE, you must first register as a user. Registration is free. Then, login and navigate to the UPLOAD tab, where you will find detailed instructions on how to use the tool. If you encounter any issues or have any questions when using PACE, please email PLOS at figures@plos.org

# Author comment: Author response to Decision Letter 3

**DOI:** 10.1371/journal.pone.0356255.r006

15 Aug 2025

Dear Dr. Ioana Gutu,

dear reviewer,

We sincerely appreciate the opportunity to resubmit our manuscript (‘Mental load in women and mothers: Causes of distress, personality traits, and psychopathology’; PONE-D-23-25403) in a revised and improved form.

The reviewer’s detailed explanations of the recommendations for improvement are highly valued, and we are grateful for this constructive guidance. We are not certain, but there may have been some misunderstandings regarding the different submitted versions, and we hope that the present revision will help to clarify these. We thank you very much for the time and effort dedicated to the evaluation of our work. In the following, we address each of the reviewer’s comments point by point.

II. Reviewer's comments:

Dear Author,

As previously suggested, your manuscript covers an interesting study area.

However, you need to carefully consider each aspect required by the reviewers, since only in this case, the research article would be considered for publication.

as previously stated,

1) the introduction needs to be improved as to better highlight the research problem, as well as the entire structure of the manuscript; maybe it is an error, but for the moment your introduction has one paragraph where you do not explain the research subject, the gap, the RQ nor a final paragraph explaining the entire manuscript structure;

Authors' response: Thank you for your valuable comment. We have clarified the research gap in two places: (1) at the end of each section in the theoretical background, and (2) in the expanded section on The Present Studies. In this section, the research gaps are summarized, the relevance of the topic is stated more explicitly, and the structure of the article is described in greater detail. We are not certain whether there may have been a misunderstanding between the different versions, but we hope that this revised version now meets the recommendations.

On page 5: “The (risk) factors associated with parental burnout may resemble those linked to mental workload. However, there is a lack of systematic scientific research examining why mental labor is perceived as stressful and how mental load relates to mental health. Gaining a precise understanding of the underlying processes and reasons why mental labor can be subjectively experienced as highly burdensome, as well as of its exact connection to mental health, is essential for developing effective strategies to reduce the burden of invisible work and promote mental well-being.”

On page 6: “Investigating the relationship between personality traits and mental load is important because certain personality factors, as described here, may act as risk factors for experiencing high levels of strain in the context of invisible work. Moreover, personality traits may interact with mental load in ways that intensify or mitigate the subjective perception of burden. Identifying such traits, and understanding both their independent effects and their interaction with mental load, can inform targeted prevention strategies, which in turn may help to reduce the burden of invisible work and the associated stressors.”

On pages 7-9:

“The Present Studies

Mental labor can be particularly stressful for women, can be associated with poorer mental health, and may have negative consequences for various domains of life (e.g., partnership and family relationships, mental health symptoms, sleep quality, and even paid employment). [8, 9, 16, 24]. In recent years, mental load has gained substantial attention in public discourse, reflecting not only its contemporary relevance but also its potential far-reaching implications across multiple domains of life, including family relationships, mental health, and occupational functioning. Despite its societal importance and the breadth of its possible consequences, mental load has received remarkably little systematic investigation within the scientific literature [24]. Given the scarcity of empirical research on mental labor, particularly with regard to its underlying mechanisms and determinants, it is crucial to systematically investigate which specific aspects of mental labor constitute the perceived burden, what role personality factors may play (e.g., as potential risk or protective factors), and how mental labor is related to psychopathological symptoms. Addressing these questions is essential, as a precise understanding of the stressors, risk factors, and their possible interactions provides the basis for developing evidence-based strategies aimed at reducing the burden of mental labor (e.g., through targeted prevention and intervention measures). By focusing on the identification of risk factors, as well as the associations between mental labor/load, personality traits, and mental health symptoms, this study seeks to address important gaps in the literature and contribute to a more comprehensive understanding of the phenomenon.

This article comprises two consecutive studies. Both studies systematically examined the perceived burden of ‘visible’ (physical) versus ‘invisible’ (cognitive/mental labor) tasks in the family context, along with the reasons underlying this burden (with Study 2 conducted in a larger sample for confirmation). Given the characteristics of mental load and its overlap with parental burnout [3, 5, 14, 16, 17, 18], it was assumed that ‘invisible’ tasks would be perceived as more burdensome than ‘visible’ tasks (hypothesis 1). Furthermore, the studies aimed to identify the specific reasons and characteristics that make mental labor burdensome. In particular, the investigation sought to confirm both the characteristics proposed in the model by Dean et al. [3] and those empirically identified to date (e.g., responsibility, time commitment) [3, 5, 16] (hypothesis 2).

In Study 1, the focus is additionally on examining the relationship between mental load and psychological symptoms (anxiety and depressive symptoms). Women and mothers experiencing high levels of overall strain (e.g., during the COVID-19 pandemic) frequently report elevated levels of burnout, loneliness, and symptoms of anxiety and depression [10, 11, 25). Such findings are consistent with biological and psychological models (see above: HPA axis, Transactional Stress Model [12]) and with previous research demonstrating that mental labor is associated with exhaustion/excessive demands, negative affect [16], and, in more recent work, depressive symptoms [24]. Against this background, it is hypothesized that medium-strength positive correlations will be found between mental load (perceived burden of ‘invisible’ tasks) and symptoms of anxiety and depression (hypothesis 3 and 4).

In Study 2, the focus is additionally on the relationship between the personality traits neuroticism as well as conscientiousness and mental labor in the context of family (traits, that are particularly relevant in the context of parental burnout, see above [20]). Individuals with higher neuroticism are considered to be more emotionally unstable, sensitive, and tend to become more easily unbalanced under stress [26]. In the context of parental burnout, there is also the assumption that neuroticism is associated with deficits in emotion regulation and that emotionally unstable parents react more strongly to life events, which can increase the likelihood of parental burnout [17]. For example, Le Vigouroux et al. [27] found that a high degree of neuroticism is a risk factor for parental burnout. So similar to findings in burnout and stress research [17, 20, 28, 29], it could also be hypothesized that neuroticism is positively related to mental load (hypothesis 5). Regarding conscientiousness, individuals with higher levels are thought to be more organized, disciplined, and have better time management [20], and negative associations have been found between conscientiousness and parental and job burnout [20, 28]. A previous study showed that low conscientiousness is a risk factor for parental burnout [27]. Accordingly, a negative correlation between conscientiousness and mental load in the context of family was assumed (hypothesis 6). Research consistently shows that single parents, especially single mothers, experience substantially higher levels of psychological distress, depressive symptoms, and burnout compared to partnered parents [10, 11, 25, 30]. In parallel, personality research has identified neuroticism and conscientiousness as predictors of parental burnout and related forms of strain [17, 27]. It seems plausible that the combination of these personality traits with the already high demands faced by single parents could further intensify the perceived burden of mental load. Based on this evidence, being a single parent was hypothesized to moderate the associations between neuroticism/conscientiousness and mental load (hypothesis 7). Since most of the mental work in the field of domestic and care work is done by women and mothers [16], above hypotheses were investigated on women and mothers.”

2) - the literature review, although you comply with the general rule of analyzing in pairs the variables, it is rather scarce, a fact reflected within your very few references.

- Please improve this part, which is also reflected within your references list.

- Please make sure that you consider and explain each variable in the light of previous literature.

Authors' response: We appreciate your suggestion that the literature could be expanded at this point. In the revised version, we have therefore incorporated additional references, particularly to enhance the clarity of the hypotheses and to strengthen their empirical foundation.

On pages 8 and 9 (see also point 1):

“In Study 1, the focus is additionally on examining the relationship between mental load and psychological symptoms (anxiety and depressive symptoms). Women and mothers experiencing high levels of overall strain (e.g., during the COVID-19 pandemic) frequently report elevated levels of burnout, loneliness, and symptoms of anxiety and depression [10, 11, 25). Such findings are consistent with biological and psychological models (see above: HPA axis, Transactional Stress Model [12]) and with previous research demonstrating that mental labor is associated with exhaustion/excessive demands, negative affect [16], and, in more recent work, depressive symptoms [24]. Against this background, it is hypothesized that medium-strength positive correlations will be found between mental load (perceived burden of ‘invisible’ tasks) and symptoms of anxiety and depression (hypothesis 3 and 4).

In Study 2, the focus is additionally on the relationship between the personality traits neuroticism as well as conscientiousness and mental labor in the context of family (traits, that are particularly relevant in the context of parental burnout, see above [20]). Individuals with higher neuroticism are considered to be more emotionally unstable, sensitive, and tend to become more easily unbalanced under stress [26]. In the context of parental burnout, there is also the assumption that neuroticism is associated with deficits in emotion regulation and that emotionally unstable parents react more strongly to life events, which can increase the likelihood of parental burnout [17]. For example, Le Vigouroux et al. [27] found that a high degree of neuroticism is a risk factor for parental burnout. So similar to findings in burnout and stress research [17, 20, 28, 29], it could also be hypothesized that neuroticism is positively related to mental load (hypothesis 5). Regarding conscientiousness, individuals with higher levels are thought to be more organized, disciplined, and have better time management [20], and negative associations have been found between conscientiousness and parental and job burnout [20, 28]. A previous study showed that low conscientiousness is a risk factor for parental burnout [27]. Accordingly, a negative correlation between conscientiousness and mental load in the context of family was assumed (hypothesis 6). Research consistently shows that single parents, especially single mothers, experience substantially higher levels of psychological distress, depressive symptoms, and burnout compared to partnered parents [10, 11, 25, 30]. In parallel, personality research has identified neuroticism and conscientiousness as predictors of parental burnout and related forms of strain [17, 27]. It seems plausible that the combination of these personality traits with the already high demands faced by single parents could further intensify the perceived burden of mental load. Based on this evidence, being a single parent was hypothesized to moderate the associations between neuroticism/conscientiousness and mental load (hypothesis 7). Since most of the mental work in the field of domestic and care work is done by women and mothers [16], above hypotheses were investigated on women and mothers.”

3)Also, from your literature review must result a number of hypotheses that you describe in the light of previous literature now, test further and make a special subchapter later and discuss the accepted/rejected hypotheses and the results in the light of previous literature.

Authors' response: We thank you for this valuable comment. As noted in our response to the previous point, the hypotheses derived from the literature review are now explicitly formulated and grounded in prior research. The Discussion section has been revised to address each hypothesis individually, indicating whether it was supported or rejected, and interpreting the results in light of previous literature.

On pages 21 and 22:

“Perceived Distribution and Burden/Load of Invisible (Mental Load) vs. Visible Daily Tasks (Hypothesis 1)…

This means that Hypothesis 1 was supported for mothers, but not for women in general. …

These findings are consistent with the conclusions of the systematic review by Reich-Stiebert et al. [16] and extend beyond the studies summarized therein by providing a direct comparison between ‘visible’ and ‘invisible’ tasks. …

These findings are consistent with the Transactional Stress Model [12] outlined in the theoretical section, as the reported burden of mental load varied substantially between individuals. Some participants indicated no burden at all, suggesting that the subjective appraisal of cognitive, invisible task (e.g., rather than their objective quantity) may be the more critical factor in determining perceived strain.”

On page 22:

“Reported Causes of Mental Load (Hypothesis 2)…

This also confirms the assumption of Dean et al. [3] that the characteristics of invisible tasks of being 'limitless' and 'continuous' in particular account for the load. The third characteristic of Dean et al. [3], the invisible (not perceiving or not acknowledging) was given by about half of the women and 2/3 of the mothers as a cause of stress.”

On page 22:

“Relationships between Mental Load and affect, anxiety, and depression (Hypotheses 3 and 4) …

We derived the hypothesized associations with generalized anxiety and depressive symptoms from studies of the association of family effort (vs. reward) and stress in mothers during the COVID-10 pandemic and anxiety and depressive symptoms [10, 11, 25]. We have now also confirmed that mental load is also positively related to generalized anxiety and depressive symptoms, which confirmed our hypotheses 3 and 4. In an earlier study [25], it was shown that mental load is associated with feelings of being overwhelmed and overburdened, which may be related to or contribute to anxiety.”

On pages 23-25:

“Personality Traits and Mental Load (Hypotheses 5-7) …

We were able to confirm the negative link between neuroticism and mental load with a moderately strong effect (hypothesis 5). …

Regarding conscientiousness, we found a positive relationship with mental load, with a very small effect, which contradicts our hypothesis 6. Le Vigouroux. et al. [27] found that low conscientiousness was associated with parental burnout (i.e., a negative association). While we referred to previous stress research (such as the study by Le Viogouroux [27]), it is conceivable that differences exist here between stress/burnout and mental load in that conscientiousness (e.g., doing tasks thoroughly, mak

# Peer review: Decision Letter 3

**DOI:** 10.1371/journal.pone.0356255.r007

**Authors:** Ioana Gutu

4 Sep 2025

Dear Dr. Jungmann,

Thank you for submitting your manuscript to PLOS ONE. After careful consideration, we feel that it has merit but does not fully meet PLOS ONE’s publication criteria as it currently stands. Therefore, we invite you to submit a revised version of the manuscript that addresses the points raised during the review process.

Please submit your revised manuscript by Oct 19 2025 11:59PM. If you will need more time than this to complete your revisions, please reply to this message or contact the journal office at plosone@plos.org. When you're ready to submit your revision, log on to https://www.editorialmanager.com/pone/ and select the 'Submissions Needing Revision' folder to locate your manuscript file.

- A rebuttal letter that responds to each point raised by the academic editor and reviewer(s). You should upload this letter as a separate file labeled 'Response to Reviewers'.
- A marked-up copy of your manuscript that highlights changes made to the original version. You should upload this as a separate file labeled 'Revised Manuscript with Track Changes'.
- An unmarked version of your revised paper without tracked changes. You should upload this as a separate file labeled 'Manuscript'.

If you would like to make changes to your financial disclosure, please include your updated statement in your cover letter. Guidelines for resubmitting your figure files are available below the reviewer comments at the end of this letter.

If applicable, we recommend that you deposit your laboratory protocols in protocols.io to enhance the reproducibility of your results. Protocols.io assigns your protocol its own identifier (DOI) so that it can be cited independently in the future. For instructions see: https://journals.plos.org/plosone/s/submission-guidelines#loc-laboratory-protocols. Additionally, PLOS ONE offers an option for publishing peer-reviewed Lab Protocol articles, which describe protocols hosted on protocols.io. Read more information on sharing protocols at https://plos.org/protocols?utm_medium=editorial-email&utm_source=authorletters&utm_campaign=protocols.

We look forward to receiving your revised manuscript.

Kind regards,

Ioana Gutu, Postdoctoral

Academic Editor

PLOS ONE

Journal Requirements:

1. If the reviewer comments include a recommendation to cite specific previously published works, please review and evaluate these publications to determine whether they are relevant and should be cited. There is no requirement to cite these works unless the editor has indicated otherwise.

[Note: HTML markup is below. Please do not edit.]

Reviewers' comments:

Reviewer's Responses to Questions

**Comments to the Author**

Reviewer #5: (No Response)

**********

2. Is the manuscript technically sound, and do the data support the conclusions?

Reviewer #5: Yes

**********

3. Has the statistical analysis been performed appropriately and rigorously?

Reviewer #5: Yes

**********

4. Have the authors made all data underlying the findings in their manuscript fully available?

The PLOS Data policy

Reviewer #5: No

**********

5. Is the manuscript presented in an intelligible fashion and written in standard English?

Reviewer #5: Yes

**********

Reviewer #5: Dear Authors,

Your Introduction has not been improved. Between all the versions, the current revision presents a manuscript with an Introduction shorter than the Abstract.

Here you have it" When thinking of family, domestic and care work, the first thing that usually comes to 47

mind are the visible tasks such as cooking, doing laundry, or driving children to their leisure 48

activities. However, these visible tasks are constantly accompanied by invisible cognitive 49

processes such as planning, organizing, and relational/emotional work. Despite increasing 50

gender equality on average, at least 75% of unpaid domestic and care work (visible and 51

invisible) is done by women and mothers [1, 2]. The invisible processes or cognitive work, 52

which are assumed to be particularly burdensome, have received increasing attention in the 53

media and society in recent years, but are scientifically under-researched [3–5]. "

Perhaps it would be better if you re-organize the manuscript and write about the main chapters Introduction Lit Rev Methodology Conclusion Future research and Limitations

Please refer to the Introduction and revise it according to the previously submitted remarks. There are numerous parts missing from it.

On the second study, the subtitle Results line 434 The descriptive statistics of the questionnaires used are shown in Table 1 has only one table and no explanation; please see to that.

i have asked you previously to clarify your Hypotheses. Please lay it down as H1: text; H2: text and so on

for the moment, you present text as " Against this background, it is hypothesized that medium-strength positive 199

correlations will be found between mental load (perceived burden of ‘invisible’ tasks) and 200

symptoms of anxiety and depression (hypothesis 3 and 4)."

this is does not respond to the previous comments and requests since it is unclear which one is H3 and which one is H4.

please refer to the previous comments from the previous review and take the problem with the utmost seriousness.

Please pay attention to the Journal requirements in regard to the citation style.

You go from Personality Traits and Mental Load to Practical implications . Maybe it would be better to explain your Results gathered for both studies, in the light of previous literature, explain if you con/in firmed the hypotheses and comment about it and further present your Conclusion future remarks and limitation.

Please consider carefully the present comments as well as the previous comments since it is the second time when i say in another words the same thing.

Please consider submitting your raw data as attached to the following version of the manuscript. Do you have the right to have and analyse it?

Best regards,

**********

what does this mean?). If published, this will include your full peer review and any attached files.

If you choose “no”, your identity will remain anonymous but your review may still be made public.

**Do you want your identity to be public for this peer review?**  For information about this choice, including consent withdrawal, please see our Privacy Policy

Reviewer #5: **Yes:** Gutu Ioana

**********

[NOTE: If reviewer comments were submitted as an attachment file, they will be attached to this email and accessible via the submission site. Please log into your account, locate the manuscript record, and check for the action link "View Attachments". If this link does not appear, there are no attachment files.]

While revising your submission, please upload your figure files to the Preflight Analysis and Conversion Engine (PACE) digital diagnostic tool, https://pacev2.apexcovantage.com/. PACE helps ensure that figures meet PLOS requirements. To use PACE, you must first register as a user. Registration is free. Then, login and navigate to the UPLOAD tab, where you will find detailed instructions on how to use the tool. If you encounter any issues or have any questions when using PACE, please email PLOS at figures@plos.org. Please note that Supporting Information files do not need this step.

# Author comment: Author response to Decision Letter 4

**DOI:** 10.1371/journal.pone.0356255.r008

5 Jan 2026

Dear Dr. Gutu,

Thank you very much for your careful re-review and for reiterating the points that still needed to be addressed. We appreciate the opportunity to improve the manuscript further. Importantly, we believe that one of the remaining issues, specifically the concern that the Introduction is too short, was due to a misunderstanding that unfortunately only became clear to us after receiving the present round of comments. In our previous revisions, we treated the full theoretical framing and conceptual development preceding the Methods as part of the Introduction/Background (i.e., everything above the Methods section). This interpretation is consistent with PLOS ONE’s general IMRAD expectations and the journal’s flexibility regarding the naming and ordering of middle sections. In addition, in preparing the revision we consulted recent PLOS ONE articles that use a concise introductory opening while integrating the broader conceptual framing within the pre-Methods section (e.g., Walsh et al., 2024; Shiely et al., 2024; Habibzadeh, 2024). Because our theoretical background (which we considered part of the Introduction) was already extensive, we initially avoided substantially lengthening the opening paragraph to prevent further manuscript expansion and to avoid redundancies.

We sincerely apologize for the confusion caused by our previous revisions. Based on both your earlier feedback and the current comments, we have revised the Introduction to make the manuscript structure explicit and easier to follow. Specifically, in the current revision we strengthened signposting at the start of the Introduction, added a brief roadmap outlining the sequence of the conceptual framing and the two studies, and ensured that the boundary between the Introduction/Background and the Methods section is unambiguous. We hope these changes resolve the misunderstanding and address your request while avoiding unnecessary duplication and further expansion of the manuscript.

Below we respond point-by-point to your comments. For transparency, we indicate where changes were made in the revised manuscript.

1. Introduction length and structure: Your Introduction has not been improved. Between all the versions, the current revision presents a manuscript with an Introduction shorter than the Abstract (copy of our Introduction). Perhaps it would be better if you re-organize the manuscript and write about the main chapters Introduction Lit Rev Methodology Conclusion Future research and Limitations. Please refer to the Introduction and revise it according to the previously submitted remarks. There are numerous parts missing from it.

Response: We clarified the manuscript structure so that it is unambiguous that the Introduction/Background comprises the full conceptual and theoretical section preceding the Methods (rather than only the opening paragraph). Specifically, we strengthened the introductory signposting and added a brief roadmap, while avoiding redundant expansion of points already covered in the theoretical background. We also aligned the overall organization with your suggestion by ensuring a clear progression from results to interpretation in light of prior literature, followed by practical implications, and ending with 'Limitations and Future Research'.

On page 3:

Introduction ‘…. Beyond these observable activities lies a largely unseen layer of ongoing thinking work: remembering, anticipating, monitoring, coordinating, and emotion/relationship management. In everyday life, this ’mental load’ can be experienced as intrusive and difficult to switch off, because it is boundaryless and recurrent [3]. The invisible processes or cognitive work have received increasing attention in the media and society in recent years (e.g., during the COVID-19 pandemic), but are scientifically under-researched, including clear conceptualizations, correlates, and mechanisms [3–5]. To address these gaps, the present manuscript first differentiates key concepts (mental labor vs. mental load), summarizes existing evidence on mental health correlates and stressful characteristics, and then reports two empirical studies that (a) compare perceived burden of invisible vs. visible tasks, (b) identify the most frequently endorsed reasons for the burden, and (c) examine associations with psychological symptoms and personality traits. In the following Literature Review, we synthesize prior work on mental load and related concepts, with a focus on its stressful characteristics and associations with mental health and personality. We then derive the aims and hypotheses of the present research before detailing the Methods.’

On page 4: ‘Literature Review’

To enhance the clarity of the manuscript structure, we adjusted the formatting of the Literature Review subheadings by using a smaller font size.

On pages 28 and 29: As recommended, we have restructured the end of the manuscript: ‘Conclusio’ after “Practical Implication’ and the manuscript ends with ‘Limitations and Future Research’

2. Comment: ‘On the second study, the subtitle Results line 434 The descriptive statistics of the questionnaires used are shown in Table 1 has only one table and no explanation; please see to that.’

Response: We agree that the sentence ‘The descriptive statistics of the questionnaires used are shown in Table 1’ required additional explanation. We have therefore expanded the Results section of Study 2 by adding a brief narrative summary of Table 1. This provides context for the subsequent correlational and regression analyses.

On page 20:

‘Descriptive statistics for Study 2 are shown in Table 1. Overall, mothers reported high levels of mental load (M = 11.63, SD = 1.81; possible range 2-14). Mean conscientiousness was 3.93 (SD = 0.63) and mean neuroticism was 3.30 (SD = 0.85; both on a 1-5 scale).’

3. Comment: ‘I have asked you previously to clarify your Hypotheses. Please lay it down as H1: text; H2: text and so on

for the moment, you present text as " Against this background, it is hypothesized that medium-strength positive 199

correlations will be found between mental load (perceived burden of ‘invisible’ tasks) and 200

symptoms of anxiety and depression (hypothesis 3 and 4)."

this is does not respond to the previous comments and requests since it is unclear which one is H3 and which one is H4.’

Response: Thank you for highlighting this again, and we apologize that our previous wording was not sufficiently clear. We agree that referring to “hypothesis 3 and 4” within a sentence can create ambiguity. In the revised manuscript, we have therefore reformatted the hypotheses as a numbered list (H1–Hn) and stated each hypothesis explicitly (e.g., “H3: …”, “H4: …”). In addition, we now clearly distinguish H3 (mental load and anxiety symptoms) from H4 (mental load and depressive symptoms), and we ensured that the same labels are used consistently throughout the Introduction, Results, and Discussion.

On page 10:

‘Hypotheses

In the following, we summarize the study hypotheses (H1-H7).

H1: Invisible tasks (mental labor) are perceived as more burdensome and less equally distributed than visible tasks, particularly among mothers.

H2: The most frequently endorsed reasons for mental load reflect key stress characteristics of mental labor such as ongoing/never finished, boundaryless/constant thinking, low appreciation, responsibility, and unequal distribution.

H3: Mental load is positively associated with symptoms of generalized anxiety.

H4: Mental load is positively associated with depressive symptoms.

H5: Mental load is positively associated with neuroticism.

H6: Mental load is negatively associated with conscientiousness.

H7: Single parent status moderates the associations between neuroticism/conscientiousness and mental load.’

4. Comment: ‘Please pay attention to the Journal requirements in regard to the citation style.’

Response: Thank you for this reminder. We apologize for the inconsistencies introduced during restructuring. We have now carefully checked the manuscript for full compliance with PLOS ONE’s reference requirements (numbered citation-sequence/Vancouver style). Specifically, we (i) corrected formatting issues in the text (including a bracket typo in one citation), (ii) ensured that all in-text citations appear as reference numbers in square brackets only (with instrument names presented outside the brackets), and (iii) revised the reference list to conform consistently to ICMJE/Vancouver formatting (author listing, journal format, and bibliographic details/DOIs where available).

5. Comment: ‘You go from Personality Traits and Mental Load to Practical implications . Maybe it would be better to explain your Results gathered for both studies, in the light of previous literature, explain if you con/in firmed the hypotheses and comment about it and further present your Conclusion future remarks and limitation.’

Response: Thank you for this helpful structural suggestion. We agree that the transition from “Personality Traits and Mental Load” to “Practical implications” was too abrupt. In the revised manuscript, we restructured the Discussion to first provide an integrated summary of the main findings from both studies, explicitly stating which hypotheses were supported, not supported, or partially supported. We then discuss the results in relation to prior literature before moving to practical implications. Finally, we added separate concluding remarks and a dedicated “Limitations and future research” section.

On page 22 (beginning of the discussion): …..Across two studies, we found that mothers experienced invisible domestic/care tasks as particularly burdensome and unequally distributed. Regarding hypothesis testing, H1 and H2 were supported (i.e., invisible tasks were rated as more burdensome than visible tasks, and mothers reported greater perceived inequality/imbalance, with effects especially pronounced among mothers). H3–H5 were supported (i.e., higher mental load was associated with higher symptoms of anxiety and depression and with higher neuroticism). H6 was not supported (conscientiousness was not negatively related to mental load but showed a small positive association). Finally, H7 was partially supported (personality moderated the mental load–symptom association for neuroticism but not for conscientiousness). In the following, we examine these findings in more detail and discuss them in light of prior literature, implications, and limitations.

The Discussion is now organized as follows:

• Begin of the discussion: Integrated summary and hypothesis confirmation paragraph (brief overview of the main findings across both studies and explicit statement of which hypotheses were supported, not supported, or partially supported)

• Burden of invisible vs. visible tasks and reasons for the burden (discussed in light of prior literature)

• Associations between mental load and psychological symptoms (discussed in light of prior literature)

• Personality traits and moderation (interpreted relative to existing evidence)

• Practical implications

• Integrated summary and conclusion

• Limitations and future research

These adjustments primarily involved relabeling section headings and reordering the existing content to ensure that results are first interpreted and contextualized before implications are presented.

6. Comment: ‘Please consider submitting your raw data as attached to the following version of the manuscript. Do you have the right to have and analyse it?’

Response: Thank you very much for this hint. We have uploaded the raw data on OSF: 10.17605/OSF.IO/NU5ZT

On page 3 in the manuscript:

‘Data availability

The anonymized datasets are available: 10.17605/OSF.IO/NU5ZT’

Thank you very much for reviewing our manuscript. We would like to apologize once again for the inconvenience and repetitions. We hope that we have now implemented the recommendations satisfactorily.

Best regards,

The authors

Attachment Submitted filename: Response letter_R4.docx

# Peer review: Decision Letter 4

**DOI:** 10.1371/journal.pone.0356255.r009

**Authors:** Marianna Mazza

15 Jun 2026

Dear Dr. Jungmann,

Thank you for submitting your manuscript to PLOS ONE. After careful consideration, we feel that it has merit but does not fully meet PLOS ONE’s publication criteria as it currently stands. Therefore, we invite you to submit a revised version of the manuscript that addresses the points raised during the review process.

Please submit your revised manuscript by Jul 30 2026 11:59PM. If you will need more time than this to complete your revisions, please reply to this message or contact the journal office at plosone@plos.org. When you're ready to submit your revision, log on to https://www.editorialmanager.com/pone/ and select the 'Submissions Needing Revision' folder to locate your manuscript file.

- A letter that responds to each point raised by the academic editor and reviewer(s). You should upload this letter as a separate file labeled 'Response to Reviewers'.
- A marked-up copy of your manuscript that highlights changes made to the original version. You should upload this as a separate file labeled 'Revised Manuscript with Track Changes'.
- An unmarked version of your revised paper without tracked changes. You should upload this as a separate file labeled 'Manuscript'.

If you would like to make changes to your financial disclosure, please include your updated statement in your cover letter. Guidelines for resubmitting your figure files are available below the reviewer comments at the end of this letter.

If applicable, we recommend that you deposit your laboratory protocols in protocols.io to enhance the reproducibility of your results. Protocols.io assigns your protocol its own identifier (DOI) so that it can be cited independently in the future. For instructions see: https://journals.plos.org/plosone/s/submission-guidelines#loc-laboratory-protocols. Additionally, PLOS ONE offers an option for publishing peer-reviewed Lab Protocol articles, which describe protocols hosted on protocols.io. Read more information on sharing protocols at https://plos.org/protocols?utm_medium=editorial-email&utm_source=authorletters&utm_campaign=protocols.

As the corresponding author, your ORCID iD is verified in the submission system and will appear in the published article. PLOS supports the use of ORCID, and we encourage all coauthors to register for an ORCID iD and use it as well. Please encourage your coauthors to verify their ORCID iD within the submission system before final acceptance, as unverified ORCID iDs will not appear in the published article. *Only*  the individual author can complete the verification step; PLOS staff *cannot*  verify ORCID iDs on behalf of authors.

We look forward to receiving your revised manuscript.

Kind regards,

Marianna Mazza

Academic Editor

PLOS One

Journal Requirements:

If the reviewer comments include a recommendation to cite specific previously published works, please review and evaluate these publications to determine whether they are relevant and should be cited. There is no requirement to cite these works unless the editor has indicated otherwise.

Please review your reference list to ensure that it is complete and correct. If you have cited papers that have been retracted, please include the rationale for doing so in the manuscript text, or remove these references and replace them with relevant current references. Any changes to the reference list should be mentioned in the rebuttal letter that accompanies your revised manuscript. If you need to cite a retracted article, indicate the article’s retracted status in the References list and also include a citation and full reference for the retraction notice.

[Note: HTML markup is below. Please do not edit.]

Reviewers' comments:

Reviewer's Responses to Questions

**Comments to the Author**

Reviewer #6: (No Response)

Reviewer #7: (No Response)

**********

2. Is the manuscript technically sound, and do the data support the conclusions?

Reviewer #6: Yes

Reviewer #7: Partly

**********

3. Has the statistical analysis been performed appropriately and rigorously?

Reviewer #6: Yes

Reviewer #7: (No Response)

**********

4. Have the authors made all data underlying the findings in their manuscript fully available?

The PLOS Data policy

Reviewer #6: No

Reviewer #7: (No Response)

**********

5. Is the manuscript presented in an intelligible fashion and written in standard English?

Reviewer #6: Yes

Reviewer #7: (No Response)

**********

Reviewer #6: The authors present an interesting and well-written manuscript that combines a literature review with two online survey studies examining mental load in women and mothers. Across the two cross-sectional studies, the authors compare the perceived burden associated with invisible versus visible tasks, identify the most commonly endorsed reasons underlying this burden, and investigate associations with psychological symptoms and personality traits. The findings are compelling and demonstrate, among other things, that women with children experience invisible tasks as more stressful than women without children, and that mothers perceive invisible everyday tasks as both more burdensome and less equally distributed than visible tasks. The manuscript has several methodological strengths, particularly the large sample sizes across both studies. I also believe that the practical implications considerably strengthen the contribution of the work. Overall, I enjoyed reading the manuscript and hope that the comments below may help further strengthen it.

Abstract: The abstract could benefit from a brief sentence describing the study design (e.g., cross-sectional online survey) and the investigated population, including a short overview of the recruitment strategy, following the sentence “Two studies aimed to systematically examine…”. It may also be helpful to include the mean age and standard deviation in parentheses alongside the sample size for each study. In addition, I would recommend briefly noting in the abstract that the manuscript also includes a literature review on mental load and related concepts.

Methods – Study 1: In the sentence “Paired t-tests were calculated to examine differences in the satisfaction of distribution and load of ‘invisible’ versus ‘visible’ work and a mixed ANOVA to additionally examine the effect of children (mothers with vs. without children)” (lines 308–311), I believe “mothers” should be replaced with “women” in the parentheses.

Results – Study 1: I wonder how were participant-generated reasons for mental load handled when they were entered in the open-response “other” category rather than selected from the predefined response options? I recommend adding a brief description.

Both studies: How long did participants take on average to complete the online surveys in Study 1 and Study 2? Were participants reimbursed for their time in any way? Additionally, was it possible for individuals to participate in both studies? I think the manuscript would benefit from these additional details.

Study 2: Given that only 7.6% of participants were single mothers, it may be worth briefly discussing whether the subgroup size provided sufficient power and stable estimates for moderation analyses involving single-parent status.

Discussion: I recommend switching the order of the final two sections so that “Limitations and Future Research” precedes “Integrated Summary and Conclusion.”

Discussion: I also recommend noting as a limitation that the present studies did not control for the number of children or whether children had special needs, as both factors may substantially influence maternal mental load.

Reviewer #7: General Comments

The authors made a considerable effort to address the concerns raised in the previous review round, and most points were adequately addressed, with the exception of question 6, which was only partially answered, and question 2, which requires minor correction. Furthermore, before the manuscript can be recommended for publication, it is necessary to clarify some points regarding the statistical approach adopted in both studies, as the current description raises doubts about transparency and methodological consistency.

Specific Comments

1. Data availability: "Do you have the right to have and analyse it?" (Previous reviewer comment #6)

The authors provided the dataset on OSF, which is appreciated. However, the reviewer's question — "Do you have the right to have and analyse it?" — was not fully addressed. The response confirms where the data are available, but does not clarify the right to use the data. This information should be explicitly stated in the manuscript. A short sentence can make this information clear (e.g., ethical approval, consent, data storage, GDPR).

2. Descriptive analyses report – study 2 (Previous reviewer comment #2)

Although the values from the descriptive analyses of the study were reported in Table (as well as in the text you included), it would have been appropriate to create a separate table for these values "within" study 2; in other words, one table for study 1 and another for study 2, each with its respective variables. This would facilitate the understanding and flow of information. In addition to avoiding repetition of information.

3. Statistical analyses — General observation (Studies 1 and 2)

Across both studies, the authors state that variables were not normally distributed, yet no specific information is provided regarding which normality test was employed (e.g., Shapiro-Wilk or Kolmogorov-Smirnov), or the outcomes of those tests. This level of detail is necessary for the reader to evaluate the appropriateness of the analytical decisions made in each study.

4. Statistical analyses — Study 1

The methods section reports the use of Paired t-tests, mixed ANOVA, and Spearman Correlations. However, given the authors' statement that the variables were not normally distributed, it is unclear whether the normality assumption was met for the parametric tests employed. This inconsistency is not addressed in either the methods or results sections.

It is acknowledged that with a large sample size (e.g., N = 354), parametric tests may be robust to normality violations in accordance with the Central Limit Theorem. However, this rationale is not explicitly stated. A clear justification for the concurrent use of parametric and non-parametric tests is necessary.

Additionally, the authors do not report whether the assumptions specific to the mixed ANOVA were examined, namely sphericity and homogeneity of variances, nor whether any corrections were applied where necessary.

Finally, the results section does not contextualise the analytical decisions made. Reporting statistical outcomes alone, without reference to the verified assumptions and the rationale for the chosen tests, limits the reproducibility and transparency of the findings.

5. Statistical analyses — Study 2

The description of the analytical strategy in Study 2 is clearer than in Study 1, and the use of the PROCESS macro (Model 1) with bootstrap confidence intervals is appropriate and adequately justified. However, two points require clarification:

The number of bootstrap samples used in the moderation analyses is not reported. The recommended standard in the literature is 5,000 bootstrap samples, and this should be explicitly stated for reproducibility.

As noted in the general observation above, the statement that variables were not normally distributed lacks specificity. Given that Spearman Correlations were chosen on this basis, identifying which variables violated normality and by which criterion they did so would strengthen the study's methodological transparency.

**********

what does this mean?). If published, this will include your full peer review and any attached files.

If you choose “no”, your identity will remain anonymous but your review may still be made public.

**Do you want your identity to be public for this peer review?**  For information about this choice, including consent withdrawal, please see our Privacy Policy

Reviewer #6: No

Reviewer #7: No

**********

[NOTE: If reviewer comments were submitted as an attachment file, they will be attached to this email and accessible via the submission site. Please log into your account, locate the manuscript record, and check for the action link "View Attachments". If this link does not appear, there are no attachment files.]

To ensure your figures meet our technical requirements, please review our figure guidelines: https://journals.plos.org/plosone/s/figures

You may also use PLOS’s free figure tool, NAAS, to help you prepare publication quality figures: https://journals.plos.org/plosone/s/figures#loc-tools-for-figure-preparation.

NAAS will assess whether your figures meet our technical requirements by comparing each figure against our figure specifications.

# Author comment: Author response to Decision Letter 5

**DOI:** 10.1371/journal.pone.0356255.r010

30 Jul 2026

Dear Dr. Marianna Mazza, dear reviewers,

We would like to sincerely thank you, as Academic Editor, and both reviewers for your continued time, effort, and thoughtful evaluation of our manuscript entitled "Mental load in women and mothers: Causes of distress, personality traits, and psychopathology" (Manuscript ID: PONE-D-23-25403R4).

We are very grateful for the constructive and insightful comments provided throughout the review process. The reviewers' suggestions have been invaluable in further improving the scientific quality, transparency, and clarity of our manuscript. We carefully considered every comment and revised the manuscript accordingly.

In this revised version, we have addressed all remaining comments raised by the reviewers. In addition to the requested methodological, statistical, and editorial revisions, we further improved the reporting of methodological procedures, ethical aspects, and data transparency. In line with the reviewers' comments and the journal's commitment to Open Science, we also revised the manuscript to provide a clearer description of data availability and participants' rights regarding the collection and use of the data. The anonymized datasets had already been publicly registered and made available via the Open Science Framework (OSF) prior to this revision, and we now describe these procedures more explicitly within the manuscript to improve transparency and reproducibility.

Below, we provide a detailed point-by-point response to every reviewer comment. For each comment, we explain how it has been addressed in the revised manuscript and indicate the corresponding locations of the changes. All modifications are highlighted in the tracked-changes version of the manuscript.

We sincerely thank you once again for your careful consideration and for the opportunity to further improve our work. We hope that the revised manuscript is now suitable for publication in PLOS ONE.

Reviewer 6 [R6]:

Reviewer #6: The authors present an interesting and well-written manuscript that combines a literature review with two online survey studies examining mental load in women and mothers. Across the two cross-sectional studies, the authors compare the perceived burden associated with invisible versus visible tasks, identify the most commonly endorsed reasons underlying this burden, and investigate associations with psychological symptoms and personality traits. The findings are compelling and demonstrate, among other things, that women with children experience invisible tasks as more stressful than women without children, and that mothers perceive invisible everyday tasks as both more burdensome and less equally distributed than visible tasks. The manuscript has several methodological strengths, particularly the large sample sizes across both studies. I also believe that the practical implications considerably strengthen the contribution of the work. Overall, I enjoyed reading the manuscript and hope that the comments below may help further strengthen it.

Authors: We sincerely thank the reviewer for the very positive and thoughtful evaluation of our manuscript. We greatly appreciate the encouraging assessment of the study, particularly the recognition of its methodological strengths, the large sample sizes, and the practical implications of the findings. We are grateful for the constructive suggestions, all of which have helped us to further improve the clarity, transparency, and quality of the manuscript. Below, we respond to each comment in detail.

1. [R6]: Abstract: The abstract could benefit from a brief sentence describing the study design (e.g., cross-sectional online survey) and the investigated population, including a short overview of the recruitment strategy, following the sentence “Two studies aimed to systematically examine…”. It may also be helpful to include the mean age and standard deviation in parentheses alongside the sample size for each study. In addition, I would recommend briefly noting in the abstract that the manuscript also includes a literature review on mental load and related concepts.

Authors: We sincerely thank the reviewer for this valuable suggestion. We agree that providing additional methodological information in the abstract improves clarity for readers. Accordingly, we have revised the abstract to explicitly state that both studies were conducted as cross-sectional online surveys, briefly describe the study populations and recruitment procedures, report the mean age and standard deviation for both samples, and indicate that the manuscript combines a literature review with two empirical studies. We believe that these revisions provide a more comprehensive overview of the study at first glance. In addition, we made minor editorial revisions at the begin of the abstract to improve clarity, conciseness, and readability.

On page 3: e.g., “…Following a literature review on mental load, two cross-sectional online survey studies systematically examined the burden of invisible (mental load) versus visible household and caregiving work, the causes of this burden, and associations with personality traits and psychological symptoms (affect, anxiety, depressiveness). Participants were recruited online via university mailing lists, social media, and parent forums. In Study 1 (N = 368 women; M = 35.98 years, SD = 8.87)…”

“…The results of Study 2 (N = 1891 mothers; M = 38.18 years, SD = 5.18)…”

Minor editorial revision at the beginning: “Mental load has received increasing public attention, particularly following societal changes such as the COVID-19 pandemic and shifts toward greater gender equality. However, despite its societal relevance, it remains comparatively under-researched. …”

2. [R6]: Methods- Study 1: In the sentence “Paired t-tests were calculated to examine differences in the satisfaction of distribution and load of ‘invisible’ versus ‘visible’ work and a mixed ANOVA to additionally examine the effect of group (mothers with vs. without children)” (lines 308–311), I believe “mothers” should be replaced with “women” in the parentheses.

Authors: We thank the reviewer for identifying this inconsistency. The wording has been corrected as suggested, and the terminology is now used consistently throughout the manuscript.

On page 14: “…Paired t-tests were calculated to examine differences in the satisfaction of distribution and load of ‘invisible’ versus ‘visible’ work and a mixed ANOVA to additionally examine the effect of children (women with vs. without children)….”

3. [R6]: Results – Study 1: I wonder how were participant-generated reasons for mental load handled when they were entered in the open-response “other” category rather than selected from the predefined response options? I recommend adding a brief description.

Authors: We thank the reviewer for this helpful suggestion. To improve methodological transparency, we have added a brief description to the Methods section explaining how responses entered in the open-text "Other" category were handled. Specifically, similar responses were reviewed and summarized into overarching categories prior to the descriptive analyses. We also added a corresponding note to Table 2 to clarify that the examples shown in parentheses represent these summarized responses.

On page 13: “…Regarding the burden, it was asked 5) how burdensome the ‘invisible’ and ‘visible’ tasks are perceived (0-100, 100 very burdensome) and 6) what exactly they perceived as stressful about the 6a) ‘invisible’ and 6b) ‘visible’ tasks using predefined multiple-choice response options (such as find nothing stressful about it, constant thoughts about it for ‘invisible’ tasks, amount/frequency, intensity/complexity/physical demands for ‘visible’ tasks, uneven distribution, low/lack of appreciation, lack of pay, etc.) and an additional open-text ‘Other’ category. Responses entered in the ’Other’ category were reviewed and summarized into overarching categories where appropriate prior to analysis.”

On page 18; Notes below Table 2: ” Responses entered in the "Other" category were summarized into overarching categories where appropriate.”

4. [R6]: Both studies: How long did participants take on average to complete the online surveys in Study 1 and Study 2? Were participants reimbursed for their time in any way? Additionally, was it possible for individuals to participate in both studies? I think the manuscript would benefit from these additional details.

Authors: We appreciate the reviewer's suggestion and have expanded the methodological description accordingly. Specifically, we now report the approximate survey duration, participant incentives, and provide additional information on the recruitment procedures. Both online surveys took approximately 15 minutes to complete. In Study 1, participants could voluntarily enter a raffle to win one of three €15 bookstore vouchers, and psychology students could receive 0.5 course credits for participation. In Study 2, no raffle or other incentive was offered (psychology students could also receive 0.5 course credits where applicable). We further clarify that, although participation in both studies cannot be completely ruled out, the studies were conducted independently and differed in their recruitment emphasis. While both studies used online recruitment, Study 2 placed a stronger emphasis on recruitment via social media platforms, making substantial overlap between the samples unlikely.

On page 11: “Completion of the online survey took approximately 15 minutes. Participants could voluntarily enter a raffle to win one of three €15 bookstore vouchers (or psychology students could receive 0.5 course credits).”

On pages 19/20: “Potential participants for the Online survey ‘Mental load in mothers’ were recruited (conducted between December 13, 2022, and January 3, 2023) via university email distribution lists and, in particular, diverse social media platforms (e.g., Instagram, X) to enhance sample heterogeneity. “ …. “Completion of the online survey took approximately 15 minutes. No raffle or other incentive was offered; however, psychology students could receive 0.5 course credits for participation where applicable.”

Limitations on page 29: “Although participation in both studies cannot be completely ruled out, the studies were conducted independently and used different recruitment emphases, particularly with a stronger focus on social media in Study 2. Therefore, substantial overlap between the samples is considered unlikely.”

5. [R6]: Study 2: Given that only 7.6% of participants were single mothers, it may be worth briefly discussing whether the subgroup size provided sufficient power and stable estimates for moderation analyses involving single-parent status.

Authors: We agree that this is an important consideration. Accordingly, we have added a brief statement to the Limitations section acknowledging that the relatively small proportion of single mothers may have reduced statistical power and the stability of estimates for moderation analyses involving single-parent status.

On page 30: “Furthermore, only a relatively small proportion of participants in Study 2 were single mothers (7.6%). Consequently, moderation analyses involving single-parent status should be interpreted with caution, as the limited subgroup size may have reduced statistical power and the stability of the corresponding estimates. Overall, it would be helpful for the generalization of the results if the samples had been more heterogeneous and representative in terms of sociodemographic variables (gender/affiliation, age/children's age, education).”

6. [R6]: Discussion: I recommend switching the order of the final two sections so that “Limitations and Future Research” precedes “Integrated Summary and Conclusion.”

Authors: We agree that placing the Limitations and Future Research section before the Integrated Summary and Conclusion improves the overall structure and readability of the Discussion. The manuscript has been revised accordingly.

7. [R6]: Discussion: I also recommend noting as a limitation that the present studies did not control for the number of children or whether children had special needs, as both factors may substantially influence maternal mental load.

Authors: Thank you for this valuable suggestion. We agree that additional family characteristics, such as the number of children and whether a child has special needs, may influence maternal mental load. We have therefore added this point to the Limitations section and recommend that future research consider these variables to better understand factors associated with mental load.

On page 30: “Future studies should also consider additional family characteristics, such as the number of children and whether a child has special needs, as these factors may further contribute to differences in maternal mental load.”

Reviewer #7: General Comments

The authors made a considerable effort to address the concerns raised in the previous review round, and most points were adequately addressed, with the exception of question 6, which was only partially answered, and question 2, which requires minor correction. Furthermore, before the manuscript can be recommended for publication, it is necessary to clarify some points regarding the statistical approach adopted in both studies, as the current description raises doubts about transparency and methodological consistency.

Authors: We thank the reviewer for the careful evaluation of our revised manuscript and for acknowledging the substantial revisions made in response to the previous review round. We appreciate the remaining comments and have carefully addressed each point below. In particular, we have clarified the statistical procedures, revised the relevant sections of the manuscript to improve methodological transparency, and corrected the issues identified in Questions 2 and 6.

Specific Comments

1. [R7]: Data availability: "Do you have the right to have and analyse it?" (Previous reviewer comment #6). The authors provided the dataset on OSF, which is appreciated. However, the reviewer's question — "Do you have the right to have and analyse it?" — was not fully addressed. The response confirms where the data are available, but does not clarify the right to use the data. This information should be explicitly stated in the manuscript. A short sentence can make this information clear (e.g., ethical approval, consent, data storage, GDPR).

Authors: Thank you for pointing out this omission. We agree that the manuscript should explicitly clarify our authorization to collect, analyze, and share the data. We have therefore revised the Methods section to state that the studies were approved by the responsible ethics committee, that all participants provided written informed consent prior to participation, including consent for the scientific use of their anonymized data, and that data handling complied with applicable data protection regulations.

On pages 11/20: “All participants provided written informed consent prior to participation, including consent for the scientific use of their anonymized data. Data were processed in accordance with applicable data protection regulations.”

2. [R7]: Descriptive analyses report – study 2 (Previous reviewer comment #2)

Although the values from the descriptive analyses of the study were reported in Table (as well as in the text you included), it would have been appropriate to create a separate table for these values "within" study 2; in other words, one table for study 1 and another for study 2, each with its respective variables. This would facilitate the understanding and flow of information. In addition to avoiding repetition of information.

Authors: Thank you for this suggestion. To improve readability and facilitate the interpretation of the results, we have separated the descriptive statistics into two tables, one for Study 1 and one for Study 2.

3., 4. And 5 [R7]: 3. Statistical analyses — General observation (Studies 1 and 2)

Across both studies, the authors state that variables were not normally distributed, yet no specific information is provided regarding which normality test was employed (e.g., Shapiro-Wilk or Kolmogorov-Smirnov), or the outcomes of those tests. This level of detail is necessary for t

Attachment Submitted filename: R5_response letter.docx

# Editor report: Decision Letter 5

**DOI:** 10.1371/journal.pone.0356255.r011

**Authors:** Marianna Mazza

2 Aug 2026

Mental load in women and mothers: Causes of distress, personality traits, and psychopathology

PONE-D-23-25403R5

Dear Dr. Jungmann,

We’re pleased to inform you that your manuscript has been judged scientifically suitable for publication and will be formally accepted for publication once it meets all outstanding technical requirements.

Within one week, you’ll receive an e-mail detailing the required amendments. When these have been addressed, you’ll receive a formal acceptance letter and your manuscript will be scheduled for publication.

An invoice will be generated when your article is formally accepted. Please note, if your institution has a publishing partnership with PLOS and your article meets the relevant criteria, all or part of your publication costs will be covered. Please make sure your user information is up-to-date by logging into Editorial Manager at Editorial Manager® and clicking the ‘Update My Information' link at the top of the page. For questions related to billing, please contact billing support.

If your institution or institutions have a press office, please notify them about your upcoming paper to help maximize its impact. If they’ll be preparing press materials, please inform our press team as soon as possible -- no later than 48 hours after receiving the formal acceptance. Your manuscript will remain under strict press embargo until 2 pm Eastern Time on the date of publication. For more information, please contact onepress@plos.org.

Kind regards,

Marianna Mazza

Academic Editor

PLOS One

Additional Editor Comments (optional):

Reviewers' comments:

# Editor report: Acceptance letter

**DOI:** 10.1371/journal.pone.0356255.r012

**Authors:** Marianna Mazza

PONE-D-23-25403R5

PLOS One

Dear Dr. Jungmann,

I'm pleased to inform you that your manuscript has been deemed suitable for publication in PLOS One. Congratulations! Your manuscript is now being handed over to our production team.

At this stage, our production department will prepare your paper for publication. This includes ensuring the following:

* All references, tables, and figures are properly cited

* All relevant supporting information is included in the manuscript submission,

* There are no issues that prevent the paper from being properly typeset

You will receive further instructions from the production team, including instructions on how to review your proof when it is ready. Please keep in mind that we are working through a large volume of accepted articles, so please give us a few days to review your paper and let you know the next and final steps.

Lastly, if your institution or institutions have a press office, please let them know about your upcoming paper now to help maximize its impact. If they'll be preparing press materials, please inform our press team within the next 48 hours. Your manuscript will remain under strict press embargo until 2 pm Eastern Time on the date of publication. For more information, please contact onepress@plos.org.

You will receive an invoice from PLOS for your publication fee after your manuscript has reached the completed accept phase. If you receive an email requesting payment before acceptance or for any other service, this may be a phishing scheme. Learn how to identify phishing emails and protect your accounts at https://explore.plos.org/phishing.

If we can help with anything else, please email us at customercare@plos.org.

Thank you for submitting your work to PLOS ONE and supporting open access.

Kind regards,

PLOS ONE Editorial Office Staff

on behalf of

Dr. Marianna Mazza

Academic Editor

PLOS One
